# Ultrasound Imaging for the Diagnosis and Evaluation of Sarcopenia: An Umbrella Review

**DOI:** 10.3390/life12010009

**Published:** 2021-12-22

**Authors:** Jia-Chi Wang, Wei-Ting Wu, Ke-Vin Chang, Lan-Rong Chen, Shao-Yu Chi, Murat Kara, Levent Özçakar

**Affiliations:** 1 Department of Physical Medicine and Rehabilitation, Taipei Veterans General Hospital, Taipei 112201, Taiwan; jcwang0726@gmail.com; 2 School of Medicine, National Yang Ming Chiao Tung University, National Yang Ming University, Taipei 112202, Taiwan; 3 Department of Physical Medicine and Rehabilitation, National Taiwan University Hospital, Bei-Hu Branch, Taipei 10845, Taiwan; wwtaustin@yahoo.com.tw (W.-T.W.); lchen@livemail.tw (L.-R.C.); shaoyuchi.tw@gmail.com (S.-Y.C.); 4 Department of Physical Medicine and Rehabilitation, National Taiwan University College of Medicine, Taipei 10048, Taiwan; 5 Center for Regional Anesthesia and Pain Medicine, Wang-Fang Hospital, Taipei Medical University, Taipei 11600, Taiwan; 6 Department of Physical and Rehabilitation Medicine, Hacettepe University Medical School, Ankara 06100, Turkey; mkaraftr@yahoo.com (M.K.); lozcakar@yahoo.com (L.Ö.)

**Keywords:** aging, muscle mass, atrophy, ultrasonography, elastography

## Abstract

There is an increasing number of reviews investigating the value of ultrasound (US) in the assessment of aging-related muscle loss. The present umbrella review aimed to systematically investigate the evidence of US imaging in the diagnosis and evaluation of sarcopenia. PubMed, Medline, Embase and Web of Science were searched from their inceptions to 31 October 2021. Systematic reviews and reviews using a systematic strategy for literature search were enrolled. The extracted data were narrated at the level of systematic reviews and meta-analyses. This umbrella review included four articles pertaining to 125 original studies and yielded several important findings. First, US is a reliable and valid imaging tool for the assessment of skeletal muscle mass. Second, among all the US parameters in B-mode, muscle thickness is the most commonly used one, which has good correlation with other standard measurements. Third, although sonoelastography and contrast-enhanced US are promising imaging modalities, their clinical utility is still limited at the current stage. Finally, a future systematic review is warranted to analyze how different ultrasonographic diagnostic criteria influence the prevalence of sarcopenia as well as its adverse health outcomes.

## 1. Introduction

Based on a meta-analysis including 58,404 participants [1], aging related decline of muscle mass and function (i.e., sarcopenia) has a prevalence of up to 10% in the general population. The reason why sarcopenia has been studied among the elderly roots from its association with several health consequences, e.g., musculoskeletal disorders [2], swallowing dysfunction [3], cognitive impairment [4] and depression [5].

Several tools have been applied for the assessment of muscle mass loss, including bioelectrical impedance analysis (BIA), magnetic resonance imaging (MRI), computed tomography (CT) and dual energy x-ray absorptiometry (DEXA). The device for BIA is low in cost and suitable for epidemiological research. While it provides an estimated body composition through the equation based on a specific population, several factors influence its accuracy, e.g., hydration status, obesity and ethnic variations [6]. The whole skeletal muscle mass can be measured by MRI with images obtained at a regular interval between the low neck and ankle. Although it was proven to be satisfactory in reproducibility and validity [7], MRI is expensive and not portable. The cross-sectional area (CSA) of the paraspinal skeletal muscles using CT has been employed for quantifying the muscle mass in patients who need abdominal surgery or evaluation of the hepatobiliary malignancy. Of note, the relevant sarcopenic index has been proven useful for the prediction of post-operative outcomes [8]. Herewith, CT requires radiation exposure and is not easily accessible. DEXA has been considered as a golden standard measurement for body composition, due to its capability of differentiating bone from soft tissue using two distinct energy spectrums [9]. Although radiation exposure in DEXA is lower than CT, its value in the prediction of adverse clinical outcomes like falls and hip fracture remains unclear [10].

On the other hand, ultrasound (US) is known to be radiation-free, cost-effective and easily portable. Using high frequency transducers, texture and size of peripheral muscles can promptly be delineated by B-mode imaging [11,12]. Additionally, some imaging techniques like sonoelastography [13] and contrast-enhanced US [14] have also been developed, enabling exploration of the mechanical properties and microcirculation of muscles with minimal invasiveness. Recently, US imaging has been incorporated into the diagnostic protocols of sarcopenia [15,16]. Likewise, there is an increasing number of reviews investigating the value of US in the assessment of aging-related muscle loss. Since the contexts of those reviews are quite diverse, the present umbrella review aimed to systematically investigate the existing evidence as regards US imaging in the diagnosis and evaluation of sarcopenia.

## 2. Methods

### 2.1. Protocol Registration

This umbrella review was conducted according to the pertinent section presented in the Cochrane Handbook and the Preferred Reporting Items for Systematic Reviews and Meta-Analysis (PRISMA) [17]. Its protocol was registered on INPLASY (International Platform of Registered Systematic Review and Meta-analysis Protocols) with a registration number of INPLASY2021110028 (https://inplasy.com/inplasy-2021-11-0028/, accessed on 20 December 2021).

### 2.2. Search Strategy

The following electronic databases were used for literature search from their inceptions to October 31, 2021: PubMed, Medline, Embase and Web of Science. The key words used for the literature search encompassed “sarcopenia”,”muscle loss”, “muscle wasting”, “muscle atrophy”, “ultrasound”, ”ultrasonography”, “sonoelastography”, “review”, “systematic review” and “meta-analysis”. The following algorithm was employed for scrutinizing potential articles: (“sarcopenia” or ”muscle loss” or “muscle wasting” or “muscle atrophy”) and (“ultrasound” or ”ultrasonography” or “sonoelastography”) and (“review”, “systematic review” or “meta-analysis”). There was no restriction of language for the literature search, the details of which are given in the Supplement Method.

### 2.3. Inclusion and Exclusion Criteria

This umbrella review included systematic reviews and meta-analyses probing the usefulness of US in the evaluation of skeletal muscles for sarcopenia. We also included reviews that had a systematic strategy for the literature search even if the authors did not specify their article type. Other inclusion criteria comprised (1) enrollment of at least one study investigating patients with sarcopenia, (2) reporting any of the following US parameters: thickness, CSA, fascicle length, pennation angle, echo intensity, strain ratio, shear modulus and shear wave velocity and (3) targeting the geriatric population (age ≥ 65 years). Reviews were excluded if they lacked (1) a systematic approach for identifying relevant articles, (2) reports of keywords for the literature search and (3) human studies. Conference proceedings, commentaries and opinion papers were also excluded.

### 2.4. Article Selection and Data Extraction

For removal of the duplicated publications, articles found in the databases were outputted to EndNote X9 (Clarivate Analytics, Philadelphia, PA, USA). Two authors (J.-C.W. and W.-T.W.) independently evaluated the titles, abstracts, and full-texts of the retrieved articles and documented the rationales for exclusion. Any discrepancy between the two reviewers was resolved by consensus after discussion or determined by the corresponding author. The following information was extracted from the eligible reviews: authors, publication year, sample size, database used for the literature search, protocol registration, target population, sample size, details of US parameters and relevant outcome.

### 2.5. Quality Assessment

Quality of the included articles was assessed by using the AMSTAR 2 (A Measurement Tool to Assess Systematic Reviews) critical appraisal tool [18]. This was performed independently by the aforementioned two authors (J.-C.W. and W.-T.W.). Again, between-rater disagreements were resolved by consensus or by the decision of the corresponding author. The AMSTAR 2 has 16 items belonging to seven critical domains (protocol registration, literature search, inclusion/exclusion criteria, risk of bias assessment, meta-analytic methods, data interpretation and publication bias). A dichotomous score (yes/no) or an ordinal scale (yes/partial yes/no) is used for rating each eligible item. Overall confidence of the reviews or meta-analyses is categorized into high, moderate, low, or critically low.

### 2.6. Data Synthesis

Extracted data were narrated at the level of systematic reviews and meta-analyses. The results were reported based on the types of US modes (B-mode vs. sonoelastography), sites of applications, reliability/validity of US measurements of muscle mass and the capability of US parameters for differentiating sarcopenia.

## 3. Results

### 3.1. Literature Search

**Figure 1:**
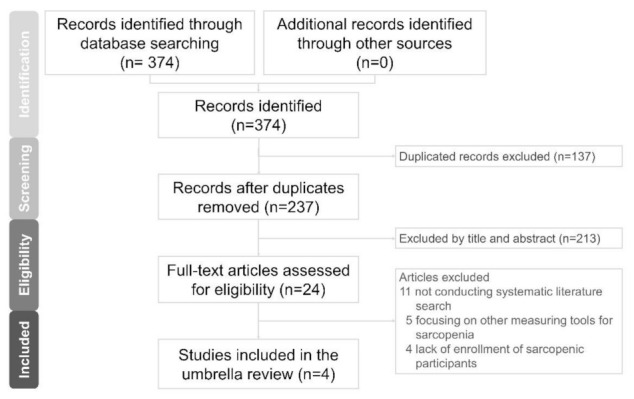
Flow diagram for literature search.

A total of 374 articles were identified from the electronic databases. Following the removal of 137 duplicates, the titles and abstracts of 237 articles were screened. Of 237 articles, 213 did not fulfill the inclusion criteria and were discarded. After full-text assessment in the remaining 24 articles, 20 were excluded with the following reasons: 11 due to not conducting systematic literature search, five due to focusing on other measuring tools for sarcopenia and four due to lack of enrollment of sarcopenic participants (Table S1). Finally, four articles were analyzed (Figure 1) [19,20,21,22].

### 3.2. Study Characteristics

The included reviews encompassed 125 non-overlapped original studies (after excluding non-human studies and study protocols) with the publication year ranging from 1993 to 2020. Features of the original studies included in each review are summarized in Tables S2–S5. The enrolled participants were healthy adults of different age ranges (young, middle and old) and patients with various complaints, diseases and clinical syndromes (low back pain, toe deformity, obesity, cardiovascular disease, diabetes, cancer, frailty and sarcopenia). The applied US modes could be categorized into B-mode, contrast-enhanced mode and sonoelastography. The sonographic parameters consisted of muscle thickness, CSA, fascicle length, pennation angle, strain ratio, shear modulus, elastic modulus, shear wave velocity and microvascular blood measurements (volume, velocity and flow). US measurement sites included muscles over the head, neck, diaphragm, abdomen, upper arm, lower arm, hand, upper leg, lower leg and foot. Details regarding the characteristics of the included reviews are provided in Table 1.

### 3.3. Methodological Quality of the Included Studies

Among the four included reviews, three were rated as of moderate quality [19,21,22] and the remaining one was rated as of low quality [20]. Only two reviews [19,22] had a pre-planned protocol prior to the study conduction, i.e., registered in an international database (PROSPERO). A complete list of excluded studies (with reasons) was not provided in any of the four reviews. In addition, none of them had conducted quantitative analysis of the retrieved data (meta-analysis). The details of quality assessment are presented in Table 2.

### 3.4. Summary of the Outcome

#### 3.4.1. Reliability and Validity of US Measurements

Reliability and validity of US in the evaluation of muscle quantity in the geriatric population were addressed in the review of Nijholt et al. [21] from 17 studies. Intra- and inter-rater reliabilities quantified by the intra-class correlation coefficient (ICC) were between −0.26 and 1.00. Higher ICC scores (between 0.72 and 1.00) were identified concerning vastus lateralis, rectus femoris, upper anterior arm and trunk muscle measurements. The validity of US had been conducted concurrently with DEXA, MRI and CT (Table S3). The corresponding r values ranged from 0.761 to 0.911. The intra-rater reliability of shear wave sonoelastography was reported to range from 0.870 to 0.978 in the review of Janczyk et al. [22].

#### 3.4.2. Sites of Muscle Measurement

The review of Perkias et al. [19] provided a detailed summary of measured muscles (upper arm, lower arm, hand, upper leg, lower leg, foot, head, neck, thorax and abdomen) as well as relevant landmarks. The review of Ticinesi et al. [20] highlighted the possibility of regional differences in the evolution of muscle mass with normal or pathological aging. The prevalence of sarcopenia was higher when it was defined as the decrease in the thigh muscle thickness as compared to the other sites. The anterior/posterior ratio of the thigh muscle was also shown to inversely correlate with age.

#### 3.4.3. US parameters in B-Mode

According to the review of Ticinesi et al. [20], muscle thickness (followed by CSA) was considered to be the simplest, quickest and most reproducible parameter for muscle mass, which correlated well with the gold standard measures. However, the associations of muscle thickness and CSA with functional parameters (e.g., grip strength and gait speed) were inconsistent. The review of Perkisas et al. [19] demonstrated the usefulness of muscle thickness to estimate the muscle volume, as “0.3 × muscle thickness + 30.5 × limb length” from the study conducted by Merrigan et al. [23].

The pennation angle, defined as the angle between the muscle fascicles and the attached aponeurosis, is commonly used to depict the architecture and strength generating capacity of the gastrocnemius muscle. The review of Ticinesi et al. [20] revealed that aging was associated with decreased pennation angles of the medial gastrocnemius but that it might not be used to differentiate sarcopenic vs. non-sarcopenic old adults. Another parameter estimated from the pennation angle is the fascicle length, whereas two formulas were described in the review of Perkisas et al. [19] from three studies [23,24,25]: fascicle length = muscle thickness × sin (pennation angle)^−1^ or = sin (aponeurosis angle + 90°) × muscle thickness/sin [180° − (aponeurosis angle +180° − pennation angle)]. Like the pennation angle, although the fascicle length tends to shorten with aging, its capability of differentiating patients with and without sarcopenia is questionable.

Increased muscle echogenicity is considered as an aging-related change owing to muscle atrophy and fatty infiltration (myosteatosis). The review of Ticinesi et al. [20] revealed higher muscle echogenicity in sarcopenic old adults or in patients with chronic obstructive pulmonary diseases as compared to young participants or age-matched healthy controls. Concerns should be raised regarding low inter- and intra-rater reproducibility of echogenicity measurements, though.

#### 3.4.4. Muscle Stiffness Measurement by Sonoelastography

The utility of sonoelastography for evaluation of sarcopenia had been investigated in two reviews. Perkisas et al. [19] highlighted the challenging points of using sonoelastography for muscle assessment as follows: differences in the type of sonoelastography (strain vs. shear wave), no cross-validation between different systems, the cost of the machine and software and not being an essential compartment of a standard US system. Janczyk et al. [22] specifically probed the issue of muscle stiffness in the geriatric population and its value for diagnosing sarcopenia. Shear wave sonoelastography was used in the majority of the included studies. They found that there were no consistent directions of muscle stiffness changes among the enrolled studies. Furthermore, one [26] of the included studies had compared muscle stiffness between the sarcopenic and non-sarcopenic populations, revealing significant between-group differences in the passive elastic constant but not in the slack elastic modulus and slack length.

#### 3.4.5. Contrast-Enhanced Assessment of Micro-Vascularity

The review of Janczyk et al. [22] shed light on the utility of contrast-enhanced US in the assessment of muscle micro-circulation. Using this technique, early capillary recruitment with dilatation of proximal vessels are seen in young adults but not in old participants, and this might imply aging-related impaired regulation of regional blood flow. The review of Perkisas et al. [19] commented that contrast-enhanced US was promising but not clinically practical, mainly due to the need for using a contrast agent.

## 4. Discussion

The present umbrella review yielded several important findings. First, US is a reliable and valid imaging tool for the assessment of skeletal muscle mass. Second, among all the B-mode parameters, muscle thickness is the most commonly used one and has good correlation with other standard measurements. Third, although sonoelastography and contrast-enhanced US are promising imaging modalities, their clinical utility is currently limited.

An umbrella review incorporates data from systematic reviews and meta-analyses, providing the highest level of evidence for clinical practice [27]. Regarding the quality of methodology, half of our included reviews had registered their protocols in an international database. The main reason would be that the PRISMA guideline was first established in 2015 [28] and updated in 2020 [17], advocating the prospective registration of systematic reviews and meta-analyses. The two included reviews [20,21] without registration were published in 2017 when the PRISMA guideline had not been prevalently adopted. Furthermore, no meta-analysis had been conducted in any of the included reviews; possibly due to their wide scope without precise definition of outcome variables and due to several US measurements across studies—making the pooled analysis extremely challenging.

US imaging has long been criticized as user-dependent and the investigators had concerns for its reliability in muscle volume evaluation. Our umbrella review identified its reliability to be acceptable for measurements of large muscles (e.g., vastus lateralis and rectus femoris) using B-mode imaging. However, the high ICC values from the included studies were obtained with strictly controlled conditions which are likely to decrease in real clinical practice [21]. In terms of validity, the parameters like muscle thickness correlated well with the other standard measurements using DEXA, MRI and CT. Nevertheless, only a few number of studies [29,30] examined the validity of US-derived equations for whole body muscle mass and the data in the geriatric population is insufficient. Whether various US parameters could be treated as substitutes for other valid measurements to diagnose sarcopenia requires further studies.

**Figure 2:**
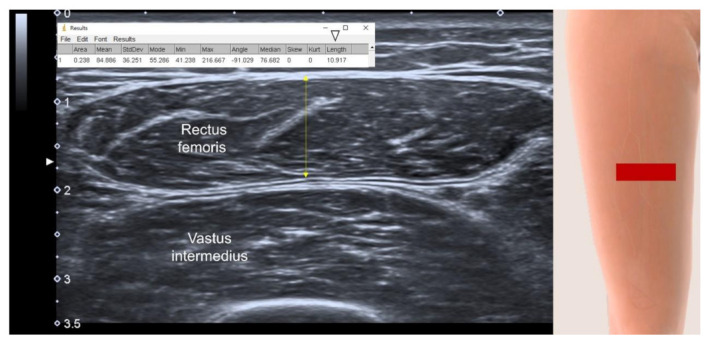
Axial US imaging shows muscle thickness measurement (double headed arrow) as the distance between the superficial and deep fasciae. White arrowhead, the value of thickness; red rectangle, illustration for the transducer’s position.

**Figure 3:**
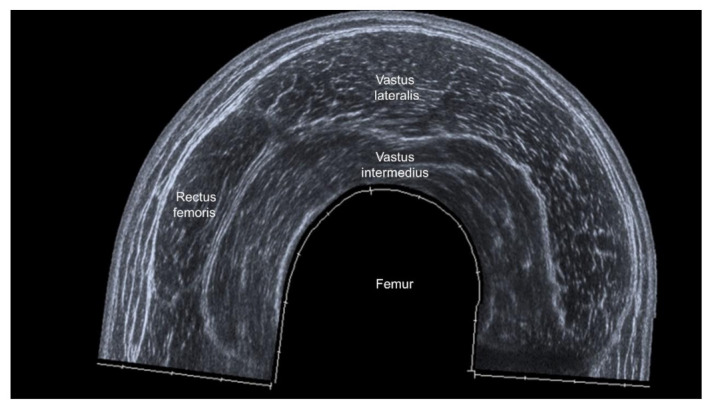
Panoramic US imaging for the anterior thigh.

The benefits of US-measured muscle thickness (Figure 2) are revealed from this umbrella review [20]. Muscle thickness can be easily defined as the maximal vertical distance between the superficial and deep fasciae [31] and it is less influenced by machine settings (e.g., signal gain, depth and focus). Herewith, the information of muscle thickness is one-dimensional and its sensitivity in detecting muscle volume loss in patients with sarcopenia can be disputed. The muscle CSA provides data from two dimensions and it is a theoretically better estimator for muscle volume than muscle thickness. However, an extended (panoramic) view (Figure 3) is usually required to obtain the whole cross-section of a large muscle [19,20,21]. As a series of images are stitched together along with the moving transducer, accuracy of the muscle length (or width) could be compromised and lead to imprecise estimation of the muscle volume. Furthermore, through reading the four included reviews, we found that three-dimensional US imaging had rarely been used for sarcopenia assessment. Compared with traditional two-dimensional B-mode imaging, three-dimensional US imaging is theoretically superior in the estimation of muscle volume, which may be developed as a future screening tool for sarcopenia before an in-depth investigation.

**Figure 4:**
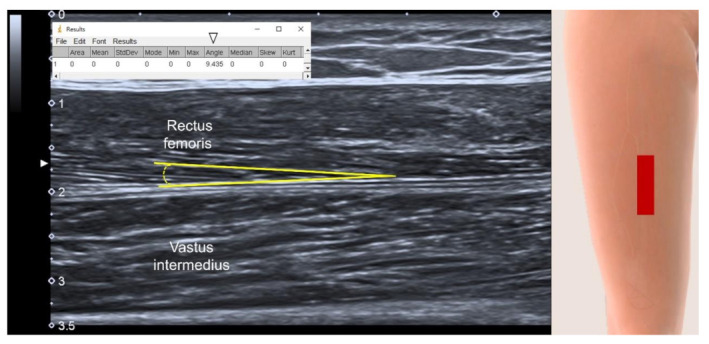
Longitudinal US imaging shows the measurement of the pennation angle (yellow lines). White arrowhead, value of the pennation angle; red rectangle, illustration for the transducer’s position.

The decrease in the pennation angle (Figure 4) results in reduction of the muscle CSA [32], which leads to compromised strength generation capacity. Although this measurement seems to be a promising parameter for the assessment of muscle volume decline, our umbrella review found that both pennation angle and fascicle length have limited values in the diagnosis of sarcopenia. The main reason would be that the pennation angle is highly influenced by the angles of adjacent joints and intensity of isometric muscle contraction [33], which naturally lead to variations during measurements due to difficulty in standardization.

**Figure 5:**
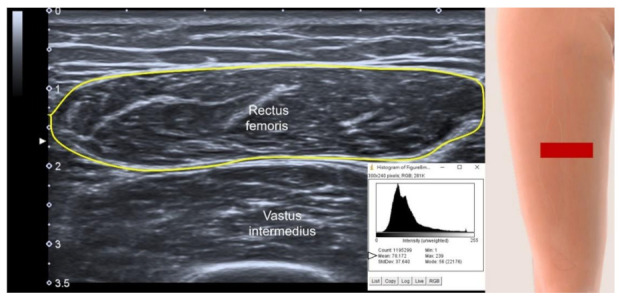
Axial US imaging shows measurement of echogenicity. Yellow circled line, region of interest; white arrowhead, the value of mean echogenicity of the histogram; red rectangle, illustration for the transducer’s position.

Muscle echogenicity (Figure 5) can be used for evaluating the extent of intra-muscular steatosis [34], which might be exacerbated in the sarcopenic population. However, this parameter is highly variable on different US machines and is also affected by the thickness of subcutaneous tissues overlying the target muscle. Furthermore, the majority of the muscles (e.g., rectus femoris) used for sarcopenia assessment have prominent intra-muscular aponeurosis which looks hyperechoic on US imaging and can cause overestimation of muscle echogenicity. All these factors would lead to poor comparability of muscle echogenicity across different individuals as well as difficulty in building standard cut-off values to define sarcopenia.

**Figure 6:**
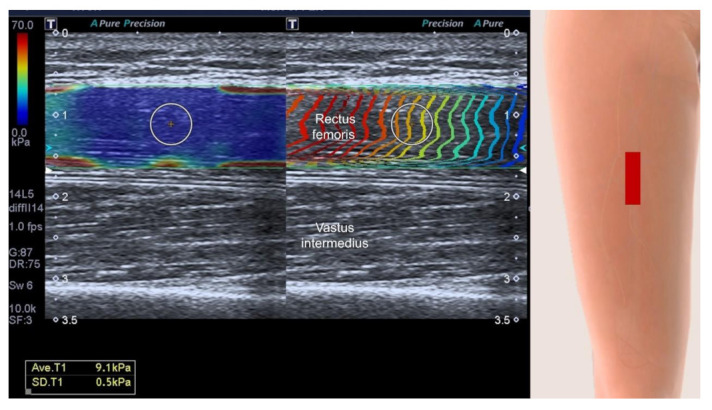
Shear wave sonoelastography shows the stiffness measurement with an average value of 9.1 kPa (standard deviation of 0.5 kPa). Yellow circle, region of interest; red rectangle, illustration for the transducer’s position.

**Figure 7:**
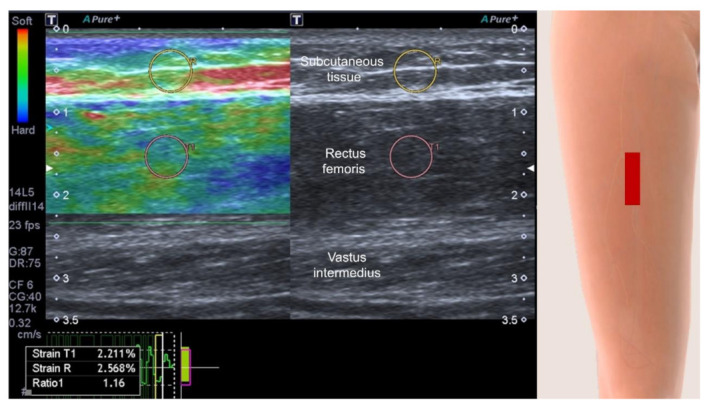
Strain sonoelastography shows the stiffness measurement with a strain ratio of 1.16. Yellow circle, region of interest for the subcutaneous tissue; pink circle, region of interest for the rectus femoris; red rectangle, illustration for the transducer’s position.

Sonoelastography is a novel imaging technique, enabling non-invasive assessment of tissue stiffness. According to this review, we identified that shear wave sonoelastography (Figure 6) had emerged as the dominant mode for stiffness measurements and that it is better than the strain mode (Figure 7) as it is less influenced by differences in manual compression force exerted on target structures [35,36]. This is also the reason why high repeatability was uncovered in the review of Janczk et al. [22]. However, even with the use of a more reliable mode, there is still no consensus whether sarcopenia softens or hardens the scanned muscle. This could also be related to non-standardization of the scanning plane (longitudinal vs. transverse) and variations in muscle fiber patterns (parallel, fusiform, unipennate and bipennate). Another possibility is the regional differences of muscle mechanical properties [22], e.g., increasing stiffness of upper limb muscles but decreasing stiffness over lower limb muscles with aging.

Although Doppler US imaging can be employed for the evaluation of macro-vascularity (without the use of contrast agents), the detection of micro-circulation at the capillary level still requires the contrast-enhanced technique [37]. Besides the injection of microbubbles to enhance the blood flow, specified hardware and software are mandatory for obtaining the parameters extracted from the time-intensity curve [14]. Therefore, the clinical utility of contrast-enhanced US imaging currently remains to be limited for the diagnosis of sarcopenia.

Our umbrella review found that none of the four reviews emphasized the cut-off values for US parameters to define low muscle mass. A few of the included studies proposed their own criteria for the diagnosis of sarcopenia. Minetto et al. [38] suggested that site-specific cut-off points of muscle thickness were 20 mm in men and 16 mm in women for rectus femoris, 17 mm in men and 15 mm in women for vastus lateralis, 23 mm in men and 22 mm in women for tibialis anterior and 13 mm in both men and women for medial gastrocnemius. Abe et al. [39] defined site-specific thigh sarcopenia using a ratio of A50/P50 MTH (anterior 50%/posterior 50% of the muscle thickness) of >2 standard deviations below the mean for young adults. We believe that another systematic review is needed to analyze how different sonographic diagnostic criteria interplay with the prevalence and adverse health outcomes of sarcopenia.

Furthermore, there was an inconsistent association between US-measured muscle mass and related physical function highlighted by the review conducted by Ticinesi et al. [20]. In the aforementioned review, some studies demonstrated a positive association between muscle mass and handgrip strength but not the gait speed [40,41]. The finding appeared understandable as the muscle mass only accounted for parts of functional measurements, like gait speed, which was also influenced by the performance of balance and neuromuscular control [42]. Furthermore, the differences in the sampling muscles across various studies for the diagnosis of sarcopenia would play a role in the aforementioned inconsistency. Recently, the International Society of Physical and Rehabilitation Medicine special interest group on sarcopenia (ISarcoPRM) included the quadriceps muscle thickness as the indicator for loss of (or low) muscle mass in the diagnostic criteria of sarcopenia [15]. They mentioned that age-related muscle loss was not uniform throughout the body and sarcopenia affected those with rich type II (fast-twitch) muscle fibers (such as the quadriceps, gastrocnemius and psoas major). Furthermore, measuring appendicular muscle mass seemed to be insufficient for detecting loss of muscle mass with aging [15,43]. As the anterior thigh muscle (i.e., quadriceps) is important for power-requiring activities such as transferring, standing, climbing, and fast walking, the use of anterior thigh muscle mass and pertinent function was strongly suggested in the diagnostic algorithm. Another reason is that anterior thigh muscle measurements (like CSA by CT and thickness by US imaging) showed higher correlations with muscle strength and performance tests when compared with appendicular/total muscle mass measurements [44]. On the other hand, anterior thigh muscle thickness could be adjusted for body mass index using the sonographic thigh adjustment ratio (STAR) because the thickness had been shown to be correlated with body mass positively and height negatively. Using the two standard deviation values below the mean from healthy young adults, the cut-off values of STAR were set at 1.4 for men and 1.0 for women [15,44].

Our umbrella review had some limitations. First, none of the four included reviews proceeded to the step of meta-analysis. Therefore, a summary statistics regarding the performance of the US parameters on the diagnosis of sarcopenia could not be generated. Second, the types of studies enrolled in each review presented a wide spectrum, comprising retrospective, cross-sectional, cohort and randomized controlled designs. Accordingly, the findings derived from the included reviews should be interpreted with caution as they are vulnerable to certain bias in methodology.

## 5. Conclusions

US imaging is a reliable and valid imaging tool for the assessment of muscle mass. US-derived muscle thickness is easily standardized and reproducible across various clinical conditions and it should therefore be considered as the primary parameter for developing the diagnostic criteria for sarcopenia. Sonoelastography and contrast-enhanced US are novel imaging techniques for the evaluation of muscle mechanical properties and physiology; however, their utility for discriminating aging- and sarcopenia-related muscle changes remains uncertain. Finally, a future systematic review is warranted to analyze how different sonographic/diagnostic criteria might relate to the prevalence of sarcopenia as well as its adverse health outcomes.

## Figures and Tables

**Table 1:** Characteristics of the included reviews.

Author, Year	Country	Protocol Registration	Included Studies (n)	Searched Database	Research Question	Ultrasound Modes	Main Conclusion
Ticinesi et al., 2017 [20]	Italy, UK	No	44	PubMed, Scopus	To review the role of muscle US for detecting muscle mass loss in older individuals	B-mode, Contrast-enhanced US	US parameters may be theoretically useful for detecting muscle mass loss and functionality in geriatric patients
Nijholt et al., 2017 [21]	Netherlands	No	17	PubMed, Cochrane, Cumulative index to Nursing and Allied Health Literature	To evaluate the reliability and validity of US for assessing muscle size in older adults	B-mode	US is a reliable and valid tool for the assessment of muscle size in older adults
Janczyk et al., 2020 [22]	France	PROSPERO (CRD42020165653)	10	Medline, Google Scholar, Scopus, SpringerLink, Science Direct	To examine whether sonoelastograpy can be a reliable method to evaluate sarcopenia in older patients	Sonoelastography (shear wave and strain modes)	No conclusion could be made about the usefulness of sonoelastograpy to assess sarcopenia due to substantial heterogenicity of actual data
Perkisas et al., 2021 [19]	Belgium, Germany, Netherlands, Spain, Poland	PROSPERO (CRD42019126106)	65	PubMed, Scopus, Web of Science	To provide standardization for the assessment of muscles of specific limbs	B-mode, Sonoelastography, Contrast-enhanced US	Different approaches for US assessment are found to likely impact the values measured

**Table 2:** Results of the AMSTAR-2 assessment.

AMSTAR-2 Item Number																
Author, Year	1	2	3	4	5	6	7	8	9	10	11	12	13	14	15	16
Ticinesi et al., 2017 [20]	N	N	Y	Y	N	N	N	N	N	N	N/A	N/A	N	N	N/A	N
Nijholt et al., 2017 [21]	Y	N	Y	Y	Y	Y	N	Y	N	N	N/A	N/A	N	N	N/A	N
Janczyk et al., 2020 [22]	Y	Y	Y	Y	Y	Y	N	Y	Y	N	N/A	N/A	Y	N	N/A	N
Perkisas et al., 2021 [19]	Y	Y	Y	Y	Y	Y	N	PY	N	N	N/A	N/A	N	N	N/A	N

Y = yes; N = no; PY = partial yes; N/A = not applicable due to absence of meta-analyses. 1 = PICO Elements; 2 = Prior Protocol; 3 = Study Designs; 4 = Search Strategy; 5 = Study Selection; 6 = Data Extraction; 7 = Excluded Studies; 8 = PICO Details; 9 = Risk of Bias Assessment; 10 Funding Sources; 11 = Meta-Analysis Methods; 12 = Risk of Bias Impact on Results; 13 = Risk of Bias Discussion; 14 = Explain Heterogeneity; 15 = Publication Bias; 16 = Conflict of Interest.

## Data Availability

Data is contained within the main text and supplementary material of the present manuscript.

## Supplementary Materials

The following supporting information can be downloaded at: https://www.mdpi.com/article/10.3390/life12010009/s1. Table S1. Excluded studies and reasons; Table S2. Studies included in the review conducted by Ticinesi et al., 2017; Table S3. Studies included in the review conducted by Nijholt et al., 2017; Table S4. Studies included in the review conducted by Janczyk et al., 2020; Table S5. Studies included in the review conducted by Perkisas et al., 2021.
