# Supplementary material for: Ultrasound Imaging for the Diagnosis and Evaluation of Sarcopenia: An Umbrella Review
Source: Life (Basel). 2021 Dec 22;12(1):9. doi: 10.3390/life12010009 (PMC8781401; doi:10.3390/life12010009)
Supplement: Supplementary file 1 [file life-12-00009-s001.zip › life-1482412-supplementary.pdf]

life

MDPI

Supplementary Material

Ultrasound Imaging for the Diagnosis and Evaluation of Sarcopenia: An Umbrella Review

Jia-Chi Wang [1,2], Wei-Ting Wu [3,4], Ke-Vin Chang [3,4,5], Lan-Rong Chen [3], Shao-Yu Chi [3], Murat Kara [6] and Levent Özçakar [6]

[1] Department of Physical Medicine and Rehabilitation, Taipei Veterans General Hospital, Taipei 112201, Taiwan; jcwang0726@gmail.com
[2] School of Medicine, National Yang Ming Chiao Tung University, National Yang Ming University, Taipei 112202, Taiwan
[3] Department of Physical Medicine and Rehabilitation, National Taiwan University Hospital, Bei-Hu Branch, Taipei 10845, Taiwan; wwtaustin@yahoo.com.tw (W-.T.W.); lchen@livemail.tw (L.-R.C.); shaoyuchi.tw@gmail.com (S.-Y.C.)
[4] Department of Physical Medicine and Rehabilitation, National Taiwan University College of Medicine, Taipei 10048, Taiwan
[5] Center for Regional Anesthesia and Pain Medicine, Wang-Fang Hospital, Taipei Medical University, Taipei 11600, Taiwan
[6] Department of Physical and Rehabilitation Medicine, Hacettepe University Medical School, Ankara 06100, Turkey; mkaraftr@yahoo.com (M.K.); lozcakar@yahoo.com (L.O.)
* Correspondence: kvchang011@gmail.com; Tel: +886223717101-5309

Supplemental Material:

Supplemental Method
Search strategy:
PubMed (update on 2021/11/02)
1. (sarcopenia[Title/Abstract]) OR (muscle loss[Title/Abstract]) OR (muscle wasting[Title/Abstract]) OR (muscle atrophy[Title/Abstract]): 24,250
2. (ultrasound[Title/Abstract]) OR (sonography[Title/Abstract]) OR (sonoelastography[Title/Abstract]): 301,264
3. (review[Title/Abstract]) OR (systematic review[Title/Abstract]) OR (meta-analysis[Title/Abstract]): 1,932,633
4. ((sarcopenia[Title/Abstract]) OR (muscle loss[Title/Abstract]) OR (muscle wasting[Title/Abstract]) OR (muscle atrophy[Title/Abstract])) AND ((ultrasound[Title/Abstract]) OR (sonography[Title/Abstract]) OR (sonoelastography[Title/Abstract])) AND ((review[Title/Abstract]) OR (systematic review[Title/Abstract]) OR (meta-analysis[Title/Abstract])): 55

Medline (update on 2021/11/02)
1. AB sarcopenia OR AB muscle loss OR AB muscle wasting OR AB muscle atrophy: 71,364
2. AB ultrasound OR AB ultrasonography OR AB sonoelastography: 314,167
3. AB review OR AB systematic review OR AB meta-analysis: 1,752,634
4. S1 AND S2 AND S3: 115

Embase (update on 2021/11/02)
1. 'sarcopenia':ab,ti OR 'muscle loss':ab,ti OR 'muscle wasting':ab,ti OR 'muscle atrophy':ab,ti: 35,244
2. 'ultrasound':ab,ti OR 'ultrasonography':ab,ti OR 'sonoelastography':ab,ti: 521,955
3. 'review':ab,ti OR 'systematic review':ab,ti OR 'meta-analysis':ab,ti: 2,388,452
4. #1 AND #2 AND #3: 83

Web of science (update on 2021/11/02)
1. AB=(sarcopenia) OR AB=(muscle loss) OR AB=(muscle wasting) OR AB=(muscle atrophy): 63,178
2. AB=(ultrasound) OR AB=(ultrasonography) OR AB=(sonoelastography): 286,842
3. AB=(review) OR AB=(systematic review) OR AB=(meta-analysis): 2,449,568
4. #1 AND #2 AND #3: 121

Table S1. Excluded studies and reasons.

Reason	Numbers	References
Not conducting systematic literature search	11	[1–11]
Focusing on other measurement tools for sarcopenia	5	[12–16]
Lack of enrollment of sarcopenic participants	5	[17–20]

Table S2. Studies included in the review conducted by Ticinesi et al., 2017.

Author(s), Year	Study Design	Sample Size	Participant's Characteristic	Ultrasound Mode	Parameter	Outcome
Abe et al., 1994 [1]	Cross-sectional	n = 117	Healthy volunteers	B-mode	Thickness (cm)	Thickness correlates with lean body mass
Naric et al., 2003 [2]	Cross-sectional	n = 30 (14 young [27–42 years], 16 older [70–81 years])	Healthy volunteers	B-mode	Length (cm), PA (°)	Smaller muscle length and PA in the elderly than the young
*Reeves et al., 2004 [3]	Cross-sectional	n = 6	Healthy volunteers	B-mode	CSA (cm^2)	Reliable CSA measurement by US; valid CSA measurement compared to MRI
Reeves et al., 2004[4]	Cross-sectional	n = 18 (9 with resistance training, 9 controls)	Healthy elderly volunteers	B-mode	Length (cm), PA (°)	Increased length and PA after resistance training
Morse et al., 2005 [5]	Cross-sectional	n = 27 (15 young, 12 elderly)	Healthy male volunteers	B-mode	Length (cm), PA (°)	No significant difference of length between the young and the elderly; smaller PA in the elderly than the young
Morse et al., 2005 [6]	Cross-sectional	n = 31 (12 young, 19 elderly)	Healthy elderly male volunteers	B-mode	Length (cm), PA (°)	No significant difference of length between the young and the elderly; smaller PA in the elderly than the young
Onambele et al., 2006 [7]	Cross-sectional	n = 70 (24 younger, 10 middle-aged, 36 older)	Healthy volunteers	B-mode	Thickness (cm)	Decreased thickness with age

Author(s), Year	Study Design	Sample Size	Participant's Characteristic	Ultrasound Mode	Parameter	Outcome
Sanada et al., 2006 [8]	Cross-sectional	n = 72	Healthy volunteers	B-mode	Thickness (cm)	Validity in thickness measurement by US for skeletal muscle mass prediction
Mian et al., 2007 [9]	Cross-sectional	n = 16 (8 young, 8 older)	Healthy volunteers	B-mode	Length (cm), PA (°)	No difference of length between the young and the older group during the stance and push-off phases
Morse et al., 2007 [10]	Cross-sectional	n =19 (11 with physical training, 8 control)	Healthy elderly male volunteers	B-mode	Length (cm), PA (°)	Increased PA after physical training
Zaidman et al., 2008 [11]	Case-control	n = 82 (37 patients, 45 healthy control)	Hereditary myopathies and healthy volunteers	B-mode	Ultrasound intensity (dB), backscatter level (dB)	Higher backscatter level in the myopathic patients than the healthy controls
Reeves et al., 2009 [12]	Case-control	n = 19 (9 conventional resistance training, 10 eccentric-only resistance training)	Healthy elderly volunteers	B-mode	Thickness (cm), length (cm), PA (°)	Increased thickness and length after both resistance training<br>Increased PA after conventional resistance training
Seymour et al., 2009 [13]	Case-control	n = 56 (30 COPD, 26 healthy)	COPD and healthy volunteers	B-mode	CSA (cm^2)	Reduced CSA in COPD compared to healthy control<br>CSA correlates with MVC strength
Atkinson et al., 2010 [14]	Randomized-controlled trial	n = 30 (16 receiving testosterone, 14 receiving placebo)	Intermediate-frail and frail elderly men	B-mode	Thickness (cm), length (cm), PA (°)	Preserved muscle thickness after testosterone treatment compared to placebo
Abe et al., 2011 [15]	Cross-sectional	n = 318	Healthy volunteers	B-mode	Thickness (cm)	Decreased thickness with age in a site-specific pattern
Abe et al., 2011 [16]	Cross-sectional	n = 1507	Healthy volunteers	B-mode	Thickness (cm)	Decreased thickness with age in a site-specific pattern
Narici et al., 2011 [17]	Case-control	n = 42 (22 with skiing training, 20 control)	Healthy elderly volunteers	B-mode	Thickness (cm), length (cm), PA (°)	Increased thickness, length and PA dafter skiing training

Author(s), Year	Study Design	Sample Size	Participant's Characteristic	Ultrasound Mode	Parameter	Outcome
Abe et al., 2012[18]	Cross-sectional	n = 53	Healthy female volunteers	B-mode	Thickness (cm)	Decreased thickness correlates with decreased walking performance
Fukumoto et al., 2012 [19]	Cross-sectional	n = 92	Healthy female volunteers	B-mode	Thickness (cm), echo intensity (AU)	Echo intensity correlates with strength
*Thomaes et al., 2012 [20]	Cross-sectional	n = 45	Older coronary artery disease patients without cardiovascular incident in the last year	B-mode	Thickness (cm)	Reliable thickness measurement by US; valid thickness measurement compared to CT
Han et al., 2013 [21]	Cross-sectional	n = 3	Healthy volunteers	B-mode	Thickness (cm)	Comparable thickness by estimation after image enhancement and by manual measurement
Mitchell et al., 2013 [22]	Cross-sectional	n = 36 (14 young, 22 older)	Healthy volunteers	B-mode, CEU	Echo intensity (dB)	Postprandial muscle microvascular responses observed in the young group but not the older
*Strasser, et al., 2013 [23]	Cross-sectional	n = 52	Healthy volunteers	B-mode	Thickness (cm), PA (°), echo intensity (AU)	Reliable thickness measurement by US
Watanabe et al., 2013 [24]	Cross-sectional	n = 184	Healthy elderly male volunteers	B-mode	Thickness (cm), echo intensity (AU)	Echo intensity correlates with strength
Abe et al., 2014 [25]	Cross-sectional	n = 1559	Healthy volunteers	B-mode	Thickness (cm)	Decreased thickness with age in a site-specific pattern
Abe et al., 2014 [26]	Cross-sectional	n = 81	Healthy middle-aged and older volunteers	B-mode	Thickness (cm)	Thickness correlates with lean mass
Abe et al., 2014 [27]	Cross-sectional	n = 1994	Healthy volunteers	B-mode	Thickness (cm)	Higher prevalence of site-specific sarcopenia (defined by a ratio of anterior/posterior thigh thickness of >2 SDs below the mean for young adults) than estimated decrease in

Author(s), Year	Study Design	Sample Size	Participant's Characteristic	Ultrasound Mode	Parameter	Outcome
						total skeletal muscle mass
Abe et al., 2014 [28]	Cross-sectional	n = 53	Healthy elder volunteers	B-mode	Thickness (cm)	Thickness correlates with handgrip strength and total skeletal muscle mass
Maddocks et al., 2014 [29]	Cross-sectional	n = 55 (20 young healthy, 18 elderly healthy, 17 COPD)	Healthy volunteers and COPD	B-mode	CSA (cm^2), echo intensity (AU)	Higher echo intensity in the COPD than the healthy
Souza et al., 2014 [30]	Randomized-controlled trial	n = 22 (12 training, 10 control)	Healthy elderly female volunteers	B-mode	Thickness (mm)	Increased thickness after training
*Takai et al., 2014 [31]	Cross-sectional	n = 77	Healthy elderly volunteers	B-mode	Thickness (cm)	Potential validity in thickness measurement by US for fat-free mass prediction
Abe et al., 2015 [32]	Cross-sectional	n = 86	Healthy young volunteers	B-mode	Thickness (cm)	Thickness correlates with handgrip strength
Abe et al., 2015 [33]	Cross-sectional	n = 79	Healthy volunteers	B-mode	Thickness (cm)	Valid muscle mass prediction by an ultrasound-based equation compared to DEXA
*Berger et al., 2015 [34]	Cross-sectional	n= 105	Healthy volunteers	B-mode	Thickness (cm), echo intensity (AU)	Thickness correlates with lean body mass by DEXA
Greening et al., 2015 [35]	Cohort	n = 191	Chronic respiratory diseases	B-mode	CSA (cm^2)	CSA correlates with readmission and death
Ido et al., 2015 [36]	Cross-sectional	n = 38	Obese adults	B-mode	Thickness (cm)	Thickness correlates with the metabolic syndrome risk
Ismail et al., 2015 [37]	Cross-sectional	n = 20	Healthy female volunteers	B-mode	Thickness (cm), echo intensity (AU)	Thickness correlates with lean body mass
Ando et al., 2016 [38]	Cross-sectional	n = 14	Healthy male volunteers	B-mode	Length (cm), PA (°)	Decreased length and increased PA during contraction
Carrilo-Esper et al., 2016 [39]	Cross-sectional	n = 109	Healthy volunteers	B-mode	Thickness (cm)	Sex-specific average diaphragmatic thickness identified

Author(s), Year	Study Design	Sample Size	Participant's Characteristic	Ultrasound Mode	Parameter	Outcome
Kuyumcu et al., 2016 [40]	Cross-sectional	n = 100	Healthy elderly volunteers	B-mode	Thickness (cm), length (cm), PA(°)	Decreased thickness (the cut-off value of 1.69 or 1.71 cm) and fascicle length (the cut-off value of 3.47 or 3.62 cm) of gastrocnemius muscles in sarcopenia (overall sensitivity > 76.92%)
Minetto et al., 2016[41]	Cross-sectional	n = 106 (60 young, 44 older)	Healthy volunteers	B-mode	Thickness (cm)	Site-specific thickness cut-points (2 SDs below the sex-specific means of the sample of young subjects) for sarcopenia identified.
Mueller et al., 2016[42]	Cohort	n = 102	Surgical intensive care unit patients	B-mode	CSA (cm^2)	CSA correlates with adverse discharge disposition
Tandon et al., 2016 [43]	Cross-sectional	n = 159	Cirrhosis	B-mode	Thickness (cm)	Thickness correlates with sarcopenia; a model combining thickness and BMI to predict sarcopenia developed

AU, arbitrary unit; BMI, body mass index; B-mode, brightness mode ultrasonography; CEU, contrast-enhanced ultrasound; COPD, chronic obstructive pulmonary disease; CSA, cross-sectional area; DEXA, double energy X-ray absorptiometry; MVC, maximal voluntary contraction; PA, pennation angle; SD, standard deviation; *, studies overlapped with those included in other reviews.

Table S3. Studies included in the review conducted by Nijholt et al., 2017.

Author(s), Year	Study Design	Sample Size	Participant's Characteristic	Ultrasound Mode	Parameter	Outcome
Sipilä et al., 1993[1]	Cross-sectional	n = 36	Healthy elderly women	B-mode	Thickness (cm), CSA (cm^2)	Valid CSA measurement by US compared to CT (r = 0.761, p < 0.001); larger CSA in the trained elderly women than the untrained control
Bemben et al., 2002[2]	Cross-sectional	n = 133 (10 young men and women, 38 postmenopausal women, 85 older men and women)	Healthy volunteers	B-mode	CSA (cm^2)	Reliable CSA measurement by US (ICC = 0.72-0.99; CV = 3.5%-6.7%)
*Reeves et al., 2004[3]	Cross-sectional	n = 6	Healthy volunteers	B-mode	CSA (cm^2)	Reliable CSA measurement by US

Author(s), Year	Study Design	Sample Size	Participant's Characteristic	Ultrasound Mode	Parameter	Outcome
						(ICC = 0.998; CV = 2.1%); valid CSA measurement compared to MRI (ICC = 0.999; mean error = 0.15 cm^2 (1.7%))
MacGillivray et al., 2009[4]	Cross-sectional	n = 11	Healthy elderly volunteers	B-mode (with 3D system)	Volume (cm^3)	Reliable volume measurement by US (ICC = 0.997 for intra-rater; ICC = 0.982 for inter-rater); valid volume measurement compared to MRI (ICC = 0.990; 95% CI of difference = -1.60 - 2.67 cm^3)
Stetts et al., 2009[5]	Cross-sectional	n = 12	Healthy elderly volunteers	B-mode	Thickness (cm)	Reliable thickness measurement by US (ICC = 0.95~1.00 for intra-image reliability; ICC = 0.77~0.97 for inter-image reliability)
Staehli et al., 2010[6]	Cross-sectional	n = 30 (10 pre-operative, 20 post-operative)	Knee osteoarthritis patients	B-mode	Thickness (cm), PA (°)	Reliable thickness measurement by US (ICC = 0.888, CV = 3.8%)
English et al., 2012[7]	Cross-sectional	n = 29	Acute stroke patients	B-mode	Thickness (cm)	Reliable thickness measurement by US (for some anatomical sites) (ICC = 0.60-1.00)
Raj et al., 2012[8]	Cross-sectional	n = 21	Healthy elderly volunteers	B-mode	Thickness (cm), fascicle length (cm), PA (°)	Reliable thickness measurement by US (ICC = 0.90-0.99)
*Thomaes et al., 2012[9]	Cross-sectional	n = 45	Older coronary artery disease patients without cardiovascular incident in the last year	B-mode	Thickness (cm)	Reliable thickness measurement by US (ICC = 0.97); valid thickness measurement compared to CT (ICC = 0.92; absolute difference between = 0.01 ± 0.12 cm)
*Strasser, et al., 2013[10]	Cross-sectional	n = 52	Healthy volunteers	B-mode	Thickness (cm), PA (°), echo intensity (AU)	Reliable thickness measurement by US (ICC = 0.85-0.97)

Author(s), Year	Study Design	Sample Size	Participant's Characteristic	Ultrasound Mode	Parameter	Outcome
Takai et al., 2013[11]	Cross-sectional	n = 77	Healthy middle-aged and older volunteers	B-mode	Thickness (cm)	Valid thickness measurement by US for predicting the skeletal muscle mass with an equation (R^2 = 0.958, SEE = 0.3 kg, % of SEE = 4.3%)
Agyapong-Badu et al., 2014[12]	Cross-sectional	n = 136	Healthy volunteers	B-mode	Thickness (cm)	Reliable thickness measurement by US (ICC = 0.88-0.89; SEM = 1.99-2.11 mm)
Cho et al., 2014[13]	Cross-sectional	n = 30	Post-stroke patients	B-mode	Thickness (cm), PA (°)	Reliable thickness and PA measurement by US (ICCs = 0.69-0.99 for intra-rater reliability; ICC = 0.70-0.99 for inter-rater reliability)
Hammond et al., 2014[14]	Cross-sectional	n = 32 (17 with COPD, 15 healthy)	COPD patients and healthy volunteers	B-mode (linear-array and curved-array transducers)	CSA (cm^2)	Reliable CSA measurement by US; (ICC = 0.79-0.99) valid CSA measurement by linear- compared to curved-array transducers (ICC = 0.982)
Sions et al., 2014[15]	Cross-sectional	n = 61	Healthy volunteers	B-mode	Thickness (cm)	Reliable thickness measurement by US (ICC = 0.84-0.94, SEM = 0.06-0.26 cm)
*Takai et al., 2014[16]	Cross-sectional	n = 77	Healthy elderly volunteers	B-mode	Thickness (cm)	Potential validity in thickness measurement by US for predicting the fat-free mass with equations (R^2 = 0.929-0.955, SEE = 2.0-2.5 kg)
*Berger et al., 2015[17]	Cross-sectional	n= 105	Healthy volunteers	B-mode	Thickness (cm), echo intensity (AU)	Thickness correlates with lean body mass assessed by DEXA (r = 0.6766-0.7448)

AU, arbitrary unit; B-mode, brightness mode ultrasonography; CI, confidence interval; COPD, chronic obstructive pulmonary disease; CSA, cross-sectional area; CT, computed tomography; CV, coefficient of variation; DEXA, dual energy X-ray absorptiometry; ICC, intraclass correlation coefficient; MRI, magnetic resonance imaging; PA, pennation angle; r, Pearson's coefficient of correlation; R^2, R-squared, coefficient of determination; SEE, standard error of the estimate; SEM, standard error of measurement; US, ultrasonography; *, studies overlapped with those included in other reviews.

Table S4. Studies included in the review conducted by Janczyk et al., 2020.

Author(s), year	Study Design	Sample Size	Participant's Characteristic	Ultrasound Mode	Parameter	Outcome
Wang et al., 2014[1]	Case-control	n = 20 (10 young female, 10 elderly female)	Healthy female volunteers	Vibro-ultrasound system	SM (kPa)	Higher SM during isometric contraction in the young female than the elderly female
Akagi et al., 2015[2]	Cross-sectional	n = 80 (31 young, 49 elderly)	Healthy volunteers	SWE	SM (kPa)	Higher SM in the young than the elderly
Eby et al., 2015[3]	Cross-sectional	n = 133 (111 younger, 32 older)	Healthy volunteers	SWE	SM (kPa)	Increased SM with age
Heizelmann et al., 2017[4]	Cross-sectional	n = 278 (254 (≤60 years), 24 (>60 years))	Healthy volunteers	SWE	SWV (m/s)	Higher SWV in the elderly than the young
Nakamura et al., 2017[5]	Cross-sectional	n = 30 (15 young, 15 elderly)	Healthy female volunteers	SWE	SM (kPa)	No significant difference of SM between the young and the elderly women
Wang et al., 2017[6]	Cross-sectional	n = 40 (10 young males, 10 young females, 10 elderly males, 10 young females)	Healthy volunteers	Vibro-ultrasound system	SM (kPa)	Higher SM during isometric contraction in the young than the elderly
Baumer et al., 2018[7]	Retrospective	n = 30 (19 asymptomatic, 11 with a supraspinatus full-thickness tear)	Asymptomatic individuals and with rotator cuff (supraspinatus full-thickness) tear	SWE	SWV (m/s)	Increased SWV with age in the asymptomatic subjects
*Alfuraih et al., 2019[8]	Cross-sectional	n = 77 (26 young, 21 middle-aged, 30 elderly)	Healthy volunteers	SWE	SWV (m/s)	Decreased SWV with age
*Saito et al., 2019[9]	Cross-sectional	n = 323 (102 young, 221 elderly)	Healthy female volunteers	Strain elastography	SR	Higher SR in the elderly women than the young women
Sun et al., 2020[10]	Cross-sectional	n = 44 (12 with muscle atrophy, 32 young healthy adults)	Young adults and patients with lower limb muscle atrophy	SWE	SM (kPa), passive elastic coefficient	Greater passive elastic coefficient in the atrophied gastrocnemius muscle than the healthy control

SM, shear modulus, elastic modulus or shear elastic modulus; SR, strain ratio; SWE, shear wave elastography; SWV, shear wave velocity; *, studies overlapped with those included in other reviews.

Table S5. Studies included in the review conducted by Perkisas et al., 2021.

Author(s), Year	Study Design	Sample Size	Participant's Characteristic	Ultrasound Mode	Parameter	Outcome
Vincent et al., 2006[1]	Cross-sectional	n = 18	Healthy volunteers	Doppler, CEU	Blood flow (ml/min), microvascular blood volume, microvascular flow velocity	Increased muscle microvascular volume after exercise and meals
Ikezoe et al., 2012[2]	Cross-sectional	n = 50 (16 young, 34 elderly)	Healthy female volunteers	B-mode	Thickness (mm)	Greater thickness in the young than the elderly
Abe et al., 2018[3]	Cross-sectional	n = 311	Healthy elderly volunteers	B-mode	Thickness (mm)	Valid prediction of fat-free adipose tissue from US compared to DEXA
Aboufazeli et al., 2018[4]	Case-control	n = 20	Healthy female volunteers	B-mode	Thickness (mm)	Reliable thickness measurement by US
Aboufazeli et al., 2018[5]	Cross-sectional	n = 60 (30 with LBP symptoms, 30 without LBP symptoms)	Female LBP patients and healthy volunteers	B-mode	Thickness (mm)	Smaller increase in thickness in the LBP patients than the healthy controls
Akagi et al., 2018[6]	Cross-sectional	n = 73 (40 young, 33 elderly)	Healthy volunteers	B-mode	Thickness (mm), echo intensity (AU)	Muscle volume estimated by thickness and echo intensity correlate with voluntary contraction
Akazawa et al., 2018[7]	Cross-sectional	n = 53 (28 chronic stroke, 25 healthy)	Chronic stroke patients and healthy volunteers	B-mode	Thickness (cm)	Smaller thickness in the chronic stroke patients than the healthy controls
Allison et al., 2018[8]	Cross-sectional	n = 50	Healthy male elderly volunteers	B-mode	Thickness (cm), length (cm), PA (°)	Increased thickness after the exercise program
Alonso-Fernandez et al., 2018[9]	Cross-sectional	n = 23	Healthy male volunteers	B-mode	Thickness (cm), length (cm), PA (°)	Increased thickness and length and decreased PA after the exercise program
Blue et al., 2018[10]	Cross-sectional	n = 44	Over-weight and obese	B-mode	CSA (cm^2), echo intensity (AU)	Increased CSA after exercise
Burton et al., 2018[11]	Cross-sectional	n = 19	Healthy volunteers	B-mode	Thickness (cm), CSA (cm^2), echo	Reliable intramuscular fat estimation by US

Author(s), Year	Study Design	Sample Size	Participant's Characteristic	Ultrasound Mode	Parameter	Outcome
					intensity (AU)	
Chang et al., 2018[12]	Cross-sectional	n = 140	Community-dwelling elderly volunteers	B-mode	Thickness (cm), echo intensity (AU)	Decreased thickness correlates with dynapenia
Cheng et al., 2018[13]	Cross-sectional	n = 40	Nasopharyngeal cancer post radiotherapy	B-mode	CSA (cm^2)	Increase in CSA correlates with hyoid displacement during swallowing
de Lima et al., 2018[14]	Cross-sectional	n = 56 (20 Hodgkin's lymphoma survivors, 36 healthy controls)	Hodgkin's lymphoma survivors and healthy volunteers	B-mode	Thickness (cm), echo intensity (AU)	No difference of thickness and echo intensity between the Hodgkin's lymphoma survivors and the healthy
Dunford et al., 2018[15]	Cross-sectional	n = 2	Healthy volunteers	CEU	Echo intensity (AU)	Microvascular flow assessed by CEU
Emami et al., 2018[16]	Cross-sectional	n = 30	Nonspecific mechanical low back pain	B-mode	Thickness (cm), CSA (cm^2)	Thickness and CSA correlate with pain and disability<br>Thickness correlates with dynamic balance
Franchi et al., 2018[17]	Cross-sectional	n = 9	Healthy young volunteers	B-mode	Thickness (cm)	Thickness correlates with CSA assessed by MRI
Fukumoto et al., 2018[18]	Cohort	n = 131	Healthy older volunteers	B-mode	Thickness (cm), echo intensity (AU)	Decreased echo intensity after higher level of physical activity
Gaffney et al., 2018[19]	Randomized-controlled trial	n = 24	Type 2 diabetes mellitus	B-mode	Thickness (cm)	Increased thickness after exercise training
Goodarzi et al., 2018[20]	Case-control	n = 40 (20 with the forward head posture, 20 with the normal head posture)	Forward head posture and normal head posture	B-mode	Thickness (cm)	No difference in thickness between forward and normal head postures
Hadda et al., 2018[21]	Cross-sectional	n = 45	Sepsis patients	B-mode	Thickness (cm)	Reliable thickness measurement by US
Harris-Love et al., 2018[22]	Cross-sectional	n = 30	Community-dwelling male volunteers	B-mode	Echo intensity (AU)	Echo intensity correlates with muscular adipose tissue estimation by CT

Author(s), Year	Study Design	Sample Size	Participant's Characteristic	Ultrasound Mode	Parameter	Outcome
Hemelryck et al., 2018[23]	Cross-sectional	n = 159 (129 rugby players, 30 healthy non-players)	Rugby players and healthy non-player control	B-mode	Thickness (cm)	Greater thickness in the rugby players than the non-players
Hida et al., 2018[24]	Cross-sectional	n = 201	Community-dwelling people	B-mode	Thickness (cm)	Decreased thickness in sarcopenia
Holtzhausen et al., 2018[25]	Cross-sectional	n = 55	Healthy volunteers	B-mode	Thickness (cm)	Thickness correlates with strength
Kajisa et al., 2018[26]	Cross-sectional	n = 90	Healthy elderly volunteers	B-mode	CSA (cm^2)	CSA correlates with the force
Kato et al., 2018[27]	Cross-sectional	n = 16	Healthy volunteers	B-mode, SWE	Thickness (mm), length (mm), SM (kPa)	Less SM decrease in exercise with compression garments
Kawai et al., 2018[28]	Cross-sectional	n = 1239	Community-dwelling older volunteers	B-mode	Thickness (mm) , echo intensity (AU)	Thickness and echo intensity correlate with percent body fat, skeletal muscle index and torque
Kim et al., 2018[29]	Cross-sectional	n = 65 (33 scoliosis, 32 healthy)	Adolescent idiopathic scoliosis and healthy	B-mode	Thickness (cm)	Thickness asymmetry in scoliosis
Kruse et al., 2018[30]	Cross-sectional	n = 12	Healthy male volunteers	B-mode	Thickness (mm), length (mm), PA (°)	Length change during limb movement correlates with heart rate and cardiac output change
Kwan et al., 2018[31]	Cross-sectional	n = 20	Healthy volunteers	B-mode	Displacement (cm)	Displacement associates with breathing
Lee et al., 2018[32]	Cross-sectional	n = 36	Healthy volunteers	B-mode	Thickness (cm)	Reliable thickness measurement by US
Magrini et al., 2018[33]	Cross-sectional	n = 35 (17 young, 18 older)	Healthy volunteers	B-mode	CSA (cm^2), Echo intensity (AU)	Decreased CSA and increased echo intensity with age
Mangum et al., 2018[34]	Cross-sectional	n = 35	Healthy volunteers	B-mode	Thickness (cm)	Different thickness change among functional movements
McKenna et al., 2018[35]	Case-control	n = 52 (26 with shoulder pain, 26 without shoulder pain)	Recreational swimmers	B-mode	Thickness (cm)	No difference in thickness with or without shoulder pain

Author(s), Year	Study Design	Sample Size	Participant's Characteristic	Ultrasound Mode	Parameter	Outcome
Merrigan et al., 2018[36]	Cross-sectional	n = 30 (15 men, 15 women)	Healthy volunteers with resistance training	B-mode	Thickness (cm), PA (°), echo intensity (AU)	Higher echo intensity in women than men
Michael et al., 2018[37]	Cross-sectional	n = 62 (31 body builders, 31 healthy)	Body builders and healthy volunteers	B-mode	Thickness (cm)	Greater thickness in the body builders than the healthy controls; thickness correlates with ulnar nerve luxation
Mickle et al., 2018[38]	Cross-sectional	n = 44 (25 with foot deformity, 19 without foot deformity)	Older adults with and without foot deformity	B-mode	Thickness (cm), CSA (cm^2)	Less thickness and CSA with foot deformity than without deformity
Misirlioglu et al., 2018[39]	Cross-sectional	n = 15	Healthy volunteers	B-mode	Thickness (mm)	Reliable thickness measurement by US
Murtagh et al., 2018[40]	Cross-sectional	n = 43 (23 elite players, 20 non-elite players)	Soccer players	B-mode	Thickness (mm), length (mm), CSA (cm^2), PA (°)	Larger CSA in the elite players than the non-elite players
Nozoe et al., 2018[41]	Cohort	n = 17	Aneurysmal subarachnoid hemorrhage	B-mode	Thickness (cm)	Decreased thickness in acute stage of aneurysmal subarachnoid hemorrhage
Ogawa et al., 2018[42]	Cross-sectional	n = 55	Elderly people referred for dysphagia assessment	B-mode	Thickness (mm), CSA (cm^2), echo intensity (AU)	Lower CSA with patients sarcopenic dysphagia than those without sarcopenic dysphagia
Park et al., 2018[43]	Cross-sectional	n = 40	Healthy adult volunteers	B-mode	Thickness (mm)	Thickness associates with facial and mandibular morphology
Poveda-Roda et al., 2018[44]	Case-control	n = 66 (31 with myofascial pain, 35 without myofascial pain)	Temporomandibular disorder with and without myofascial pain involving the masseter muscle	B-mode	Width (mm)	No difference in width between those with and those without myofascial pain
Silva et al., 2018[45]	Cross-sectional	n = 17	Type 2 diabetes mellitus	B-mode	Thickness (mm), CSA (cm^2), PA (°)	Reliable thickness and CSA measurement by US
Sterczala et al., 2018[46]	Cross-sectional	n = 24	Healthy volunteers	B-mode	CSA (cm^2), echo	Greater echo intensity in the older than the young

Author(s), Year	Study Design	Sample Size	Participant's Characteristic	Ultrasound Mode	Parameter	Outcome
		(14 young, 10 older)			intensity (AU)	
Sutherlin et al., 2018[47]	Cross-sectional	n = 59 (25 with history of low back pain, 24 without history of low back pain)	With or without history of low back pain	B-mode	Thickness (cm)	Less thickness changes across positions with history of low back pain than without history of low back pain
Watanabe et al., 2018[48]	Cross-sectional	n = 40	Healthy volunteers	B-mode	Echo intensity (AU)	Echo intensity correlates with muscle attenuation assessed by CT
Yoshiko et al., 2018[49]	Cross-sectional	n = 22	Healthy elderly volunteers	B-mode	Echo intensity (AU)	Echo intensity correlates with muscle mass and performance
Akazawa et al., 2019[50]	Cross-sectional	n = 72	Chronic stroke	B-mode	Thickness (cm), echo intensity (AU)	Less thickness and higher echo intensity with lower body mass index
*Alfuraih et al., 2019[51]	Cross-sectional	n = 77 (26 young, 21 middle-aged, and 30 elderly)	Healthy volunteers	SWE	SWV (m/s)	Decreased SWV with age
Aubertin-Leheudre et al., 2019[52]	Cross-sectional	n = 44	Hospitalized elderly patients	B-mode	Thickness (mm), PA (°)	Thickness and PA correlate with disability
Bartolomei et al., 2019[53]	Cross-sectional	n = 20	Resistance-trained men	B-mode	Thickness (mm), length (mm), PA (°)	Length correlates with peak force
Bey et al., 2019[54]	Cohort	n = 34 (19 pregnant, 15 non-pregnant)	Pregnant and non-pregnant women	B-mode	Thickness (mm), PA (°)	Increased thickness and PA in the late stage of pregnancy
Carletti et al., 2019[55]	Cross-sectional	n = 15	Edentulous elderly volunteers	B-mode	Thickness (cm)	Increased thickness after using new complete dentures
Chantaramanee et al., 2019[56]	Cross-sectional	n = 94	Healthy elderly volunteers	B-mode	Thickness (mm), echo intensity (AU)	Echo intensity correlates with thickness and oral diadochokinesis
Cuellar et al., 2019[57]	Randomized-controlled trial	n = 186 (95 with Vitamin D supplementation, 91 using placebo)	Knee osteoarthritis	B-mode	Thickness (cm)	No change in thickness after vitamin D supplementation

Author(s), Year	Study Design	Sample Size	Participant's Characteristic	Ultrasound Mode	Parameter	Outcome
Hasegawa et al., 2019[58]	Cross-sectional	n = 73	Elderly	B-mode	Thickness (mm)	Reliable thickness measurement by US Thickness correlates with muscle mass
Lopez et al., 2019[59]	Cross-sectional	n = 22	Healthy elderly volunteers	B-mode	Thickness (mm), CSA (mm^2), echo intensity (AU)	No difference in thickness, CSA and echo intensity measurement during resting time after postural change
*Saito et al., 2019[60]	Cross-sectional	n = 323 (102 young, 221 older)	Healthy female volunteers	B-mode, strain elastography	Thickness (mm), strain ratio	Less thickness and higher strain ratio in the elderly than the young
Shah et al., 2019[61]	Cross-sectional	n = 65	Elderly trauma patients	B-mode	Thickness (cm)	Thickness correlates with frailty
Šimunič et al., 2019[62]	Cohort	n = 10	Healthy male volunteers	B-mode	Thickness (mm), PA (°)	Decreased thickness and PA after bed rest

AU, arbitrary unit; B-mode, brightness mode ultrasonography; CEU, contrast-enhanced ultrasound; CSA, cross-sectional area; CT, computed tomography; DEXA, double; energy X-ray absorptiometry; MRI, magnetic resonance imaging; PA, pennation angle; SM, shear modulus, elastic modulus or shear elastic modulus; SWE, shear wave elastography; US, ultrasonography; *, studies overlapped with those included in other reviews.

PRISMA Checklist

Section and Topic	Item #	Checklist item	Location where item is reported
**TITLE**			
Title	1	Identify the report as a systematic review.	P1
**ABSTRACT**			
Abstract	2	See the PRISMA 2020 for Abstracts checklist.	P1
**INTRODUCTION**			
Rationale	3	Describe the rationale for the review in the context of existing knowledge.	P2
Objectives	4	Provide an explicit statement of the objective(s) or question(s) the review addresses.	P2
**METHODS**			
Eligibility criteria	5	Specify the inclusion and exclusion criteria for the review and how studies were grouped for the syntheses.	P3
Information sources	6	Specify all databases, registers, websites, organisations, reference lists and other sources searched or consulted to identify studies. Specify the date when each source was last searched or consulted.	P3
Search strategy	7	Present the full search strategies for all databases, registers and websites, including any filters and limits used.	P2
Selection process	8	Specify the methods used to decide whether a study met the inclusion criteria of the review, including how many reviewers screened each record and each report retrieved, whether they worked independently, and if applicable, details of automation tools used in the process.	P3
Data collection process	9	Specify the methods used to collect data from reports, including how many reviewers collected data from each report, whether they worked independently, any processes for obtaining or confirming data from study investigators, and if applicable, details of automation tools used in the process.	P3
Data items	10a	List and define all outcomes for which data were sought. Specify whether all results that were compatible with each outcome domain in each study were sought (e.g. for all measures, time points, analyses), and if not, the methods used to decide which results to collect.	P3
	10b	List and define all other variables for which data were sought (e.g. participant and intervention characteristics, funding sources). Describe any assumptions made about any missing or unclear information.	P3
Study risk of bias assessment	11	Specify the methods used to assess risk of bias in the included studies, including details of the tool(s) used, how many reviewers assessed each study and whether they worked independently, and if applicable, details of automation tools used in the process.	P3
Effect measures	12	Specify for each outcome the effect measure(s) (e.g. risk ratio, mean difference) used in the synthesis or presentation of results.	P3
Synthesis methods	13a	Describe the processes used to decide which studies were eligible for each synthesis (e.g. tabulating the study	P4

Section and Topic	Item #	Checklist item	Location where item is reported
		intervention characteristics and comparing against the planned groups for each synthesis (item #5)).	
	13b	Describe any methods required to prepare the data for presentation or synthesis, such as handling of missing summary statistics, or data conversions.	P4
	13c	Describe any methods used to tabulate or visually display results of individual studies and syntheses.	N.A.
	13d	Describe any methods used to synthesize results and provide a rationale for the choice(s). If meta-analysis was performed, describe the model(s), method(s) to identify the presence and extent of statistical heterogeneity, and software package(s) used.	N.A.
	13e	Describe any methods used to explore possible causes of heterogeneity among study results (e.g. subgroup analysis, meta-regression).	N.A.
	13f	Describe any sensitivity analyses conducted to assess robustness of the synthesized results.	N.A.
Reporting bias assessment	14	Describe any methods used to assess risk of bias due to missing results in a synthesis (arising from reporting biases).	P6
Certainty assessment	15	Describe any methods used to assess certainty (or confidence) in the body of evidence for an outcome.	P7
**RESULTS**			
Study selection	16a	Describe the results of the search and selection process, from the number of records identified in the search to the number of studies included in the review, ideally using a flow diagram.	P4
	16b	Cite studies that might appear to meet the inclusion criteria, but which were excluded, and explain why they were excluded.	P4
Study characteristics	17	Cite each included study and present its characteristics.	P5
Risk of bias in studies	18	Present assessments of risk of bias for each included study.	P6
Results of individual studies	19	For all outcomes, present, for each study: (a) summary statistics for each group (where appropriate) and (b) an effect estimate and its precision (e.g. confidence/credible interval), ideally using structured tables or plots.	P7
Results of syntheses	20a	For each synthesis, briefly summarise the characteristics and risk of bias among contributing studies.	P7
	20b	Present results of all statistical syntheses conducted. If meta-analysis was done, present for each the summary estimate and its precision (e.g. confidence/credible interval) and measures of statistical heterogeneity. If comparing groups, describe the direction of the effect.	N.A.
	20c	Present results of all investigations of possible causes of heterogeneity among study results.	N.A.
	20d	Present results of all sensitivity analyses conducted to assess the robustness of the synthesized results.	N.A.
Reporting biases	21	Present assessments of risk of bias due to missing results (arising from reporting biases) for each synthesis assessed.	P7

Section and Topic	Item #	Checklist item	Location where item is reported
Certainty of evidence	22	Present assessments of certainty (or confidence) in the body of evidence for each outcome assessed.	P7
**DISCUSSION**			
Discussion	23a	Provide a general interpretation of the results in the context of other evidence.	P 9-13
	23b	Discuss any limitations of the evidence included in the review.	P 9-13
	23c	Discuss any limitations of the review processes used.	P 9-13
	23d	Discuss implications of the results for practice, policy, and future research.	P 9-13
**OTHER INFORMATION**			
Registration and protocol	24a	Provide registration information for the review, including register name and registration number, or state that the review was not registered.	P2
	24b	Indicate where the review protocol can be accessed, or state that a protocol was not prepared.	P2
	24c	Describe and explain any amendments to information provided at registration or in the protocol.	P2
Support	25	Describe sources of financial or non-financial support for the review, and the role of the funders or sponsors in the review.	P14
Competing interests	26	Declare any competing interests of review authors.	P14
Availability of data, code and other materials	27	Report which of the following are publicly available and where they can be found: template data collection forms; data extracted from included studies; data used for all analyses; analytic code; any other materials used in the review.	P14

References

1. Kara, M.; Kaymak, B.; Frontera, W.; Ata, A.M.; Ricci, V.; Ekiz, T.; Chang, K.V.; Han, D.S.; Michail, X.; Quittan, M., et al. Diagnosing sarcopenia: Functional perspectives and a new algorithm from the ISarcoPRM. *J Rehabil Med* **2021**, *53*, jrm00209, doi:10.2340/16501977-2851.
2. Fernández-Pombo, A.; Rodríguez-Carnero, G.; Castro, A.I.; Cantón-Blanco, A.; Seoane, L.M.; Casanueva, F.F.; Crujeiras, A.B.; Martínez-Olmos, M.A. Relevance of nutritional assessment and treatment to counteract cardiac cachexia and sarcopenia in chronic heart failure. *Clin Nutr* **2021**, *40*, 5141-5155, doi:10.1016/j.clnu.2021.07.027.
3. Ponti, F.; De Cinque, A.; Fazio, N.; Napoli, A.; Guglielmi, G.; Bazzocchi, A. Ultrasound imaging, a stethoscope for body composition assessment. *Quant Imaging Med Surg* **2020**, *10*, 1699-1722, doi:10.21037/qims-19-1048.
4. Mirón Mombiela, R.; Vucetic, J.; Rossi, F.; Tagliafico, A.S. Ultrasound Biomarkers for Sarcopenia: What Can We Tell So Far? *Semin Musculoskelet Radiol* **2020**, *24*, 181–193, doi:10.1055/s-0039-3402745.
5. Abe, T.; Kondo, M.; Kawakami, Y.; Fukunaga, T. Prediction equations for body composition of Japanese adults by B-mode ultrasound. *American journal of human biology : the official journal of the Human Biology Council* **1994**, *6*, 161–170, doi:10.1002/ajhb.1310060204.
6. Narici, M.V.; Maganaris, C.N.; Reeves, N.D.; Capodaglio, P. Effect of aging on human muscle architecture. *Journal of applied physiology (Bethesda, Md. : 1985)* **2003**, *95*, 2229–2234, doi:10.1152/japplphysiol.00433.2003.
7. Reeves, N.D.; Maganaris, C.N.; Narici, M.V. Ultrasonographic assessment of human skeletal muscle size. *European journal of applied physiology* **2004**, *91*, 116–118, doi:10.1007/s00421–003-0961-9.
8. Reeves, N.D.; Narici, M.V.; Maganaris, C.N. Effect of resistance training on skeletal muscle-specific force in elderly humans. *Journal of applied physiology (Bethesda, Md. : 1985)* **2004**, *96*, 885–892, doi:10.1152/japplphysiol.00688.2003.
9. Morse, C.I.; Thom, J.M.; Birch, K.M.; Narici, M.V. Changes in triceps surae muscle architecture with sarcopenia. *Acta physiologica Scandinavica* **2005**, *183*, 291–298, doi:10.1111/j.1365-201X.2004.01404.x.
10. Morse, C.I.; Thom, J.M.; Reeves, N.D.; Birch, K.M.; Narici, M.V. In vivo physiological cross-sectional area and specific force are reduced in the gastrocnemius of elderly men. *Journal of applied physiology (Bethesda, Md. : 1985)* **2005**, *99*, 1050–1055, doi:10.1152/japplphysiol.01186.2004.
11. Onambele, G.L.; Narici, M.V.; Maganaris, C.N. Calf muscle-tendon properties and postural balance in old age. *Journal of applied physiology (Bethesda, Md. : 1985)* **2006**, *100*, 2048-2056, doi:10.1152/japplphysiol.01442.2005.
12. Sanada, K.; Kearns, C.F.; Midorikawa, T.; Abe, T. Prediction and validation of total and regional skeletal muscle mass by ultrasound in Japanese adults. *European journal of applied physiology* **2006**, *96*, 24–31, doi:10.1007/s00421-005-0061-0.
13. Mian, O.S.; Thom, J.M.; Ardigò, L.P.; Minetti, A.E.; Narici, M.V. Gastrocnemius muscle-tendon behaviour during walking in young and older adults. *Acta physiologica (Oxford, England)* **2007**, *189*, 57-65, doi:10.1111/j.1748-1716.2006.01634.x.
14. Morse, C.I.; Thom, J.M.; Mian, O.S.; Birch, K.M.; Narici, M.V. Gastrocnemius specific force is increased in elderly males following a 12-month physical training programme. *European journal of applied physiology* **2007**, *100*, 563-570, doi:10.1007/s00421-006-0246-1.
15. Zaidman, C.M.; Holland, M.R.; Anderson, C.C.; Pestronk, A. Calibrated quantitative ultrasound imaging of skeletal muscle using backscatter analysis. *Muscle & nerve* **2008**, *38*, 893-898, doi:10.1002/mus.21052.
16. Reeves, N.D.; Maganaris, C.N.; Longo, S.; Narici, M.V. Differential adaptations to eccentric versus conventional resistance training in older humans. *Experimental physiology* **2009**, *94*, 825-833, doi:10.1113/expphysiol.2009.046599.
17. Seymour, J.M.; Ward, K.; Sidhu, P.S.; Puthucheary, Z.; Steier, J.; Jolley, C.J.; Rafferty, G.; Polkey, M.I.; Moxham, J. Ultrasound measurement of rectus femoris cross-sectional area and the relationship with quadriceps strength in COPD. *Thorax* **2009**, *64*, 418, doi:10.1136/thx.2008.103986.
18. Atkinson, R.A.; Srinivas-Shankar, U.; Roberts, S.A.; Connolly, M.J.; Adams, J.E.; Oldham, J.A.; Wu, F.C.; Seynnes, O.R.; Stewart, C.E.; Maganaris, C.N., et al. Effects of testosterone on skeletal muscle architecture in intermediate-frail and frail elderly men. *The journals of gerontology. Series A, Biological sciences and medical sciences* **2010**, *65*, 1215-1219, doi:10.1093/gerona/glq118.
19. Abe, T.; Kawakami, Y.; Kondo, M.; Fukunaga, T. Comparison of ultrasound-measured age-related, site-specific muscle loss between healthy Japanese and German men. *Clinical physiology and functional imaging* **2011**, *31*, 320–325, doi:10.1111/j.1475-097X.2011.01021.x.
20. Abe, T.; Sakamaki, M.; Yasuda, T.; Bemben, M.G.; Kondo, M.; Kawakami, Y.; Fukunaga, T. Age-related, site-specific muscle loss in 1507 Japanese men and women aged 20 to 95 years. *Journal of sports science & medicine* **2011**, *10*, 145–150.
21. Narici, M.V.; Flueck, M.; Koesters, A.; Gimpl, M.; Reifberger, A.; Seynnes, O.R.; Niebauer, J.; Rittweger, J.; Mueller, E. Skeletal muscle remodeling in response to alpine skiing training in older individuals. *Scandinavian journal of medicine & science in sports* **2011**, *21 Suppl 1*, 23-28, doi:10.1111/j.1600-0838.2011.01338.x.
22. Abe, T.; Ogawa, M.; Loenneke, J.P.; Thiebaud, R.S.; Loftin, M.; Mitsukawa, N. Relationship between site-specific loss of thigh muscle and gait performance in women: the HIREGASAKI study. *Arch Gerontol Geriatr* **2012**, *55*, e21-25, doi:10.1016/j.archger.2012.06.009.
23. Fukumoto, Y.; Ikezoe, T.; Yamada, Y.; Tsukagoshi, R.; Nakamura, M.; Mori, N.; Kimura, M.; Ichihashi, N. Skeletal muscle quality assessed from echo intensity is associated with muscle strength of middle-aged and elderly persons. *European journal of applied physiology* **2012**, *112*, 1519-1525, doi:10.1007/s00421-011-2099-5.

24. Thomaes, T.; Thomis, M.; Onkelinx, S.; Coudyzer, W.; Cornelissen, V.; Vanhees, L. Reliability and validity of the ultrasound technique to measure the rectus femoris muscle diameter in older CAD-patients. *BMC medical imaging* **2012**, *12*, 7, doi:10.1186/1471-2342-12-7.
25. Han, P.; Chen, Y.; Ao, L.; Xie, G.; Li, H.; Wang, L.; Zhou, Y. Automatic thickness estimation for skeletal muscle in ultrasonography: evaluation of two enhancement methods. *Biomedical engineering online* **2013**, *12*, 6, doi:10.1186/1475-925x-12-6.
26. Mitchell, W.K.; Phillips, B.E.; Williams, J.P.; Rankin, D.; Smith, K.; Lund, J.N.; Atherton, P.J. Development of a new Sonovue™ contrast-enhanced ultrasound approach reveals temporal and age-related features of muscle microvascular responses to feeding. *Physiological reports* **2013**, *1*, e00119, doi:10.1002/phy2.119.
27. Strasser, E.M.; Draskovits, T.; Praschak, M.; Quittan, M.; Graf, A. Association between ultrasound measurements of muscle thickness, pennation angle, echogenicity and skeletal muscle strength in the elderly. *Age (Dordrecht, Netherlands)* **2013**, *35*, 2377-2388, doi:10.1007/s11357-013-9517-z.
28. Watanabe, Y.; Yamada, Y.; Fukumoto, Y.; Ishihara, T.; Yokoyama, K.; Yoshida, T.; Miyake, M.; Yamagata, E.; Kimura, M. Echo intensity obtained from ultrasonography images reflecting muscle strength in elderly men. *Clinical interventions in aging* **2013**, *8*, 993-998, doi:10.2147/cia.S47263.
29. Abe, T.; Loenneke, J.P.; Thiebaud, R.S.; Fukunaga, T. Age-related site-specific muscle wasting of upper and lower extremities and trunk in Japanese men and women. *Age (Dordrecht, Netherlands)* **2014**, *36*, 813-821, doi:10.1007/s11357-013-9600-5.
30. Abe, T.; Patterson, K.M.; Stover, C.D.; Geddam, D.A.; Tribby, A.C.; Lajza, D.G.; Young, K.C. Site-specific thigh muscle loss as an independent phenomenon for age-related muscle loss in middle-aged and older men and women. *Age (Dordrecht, Netherlands)* **2014**, *36*, 9634, doi:10.1007/s11357-014-9634-3.
31. Abe, T.; Thiebaud, R.S.; Loenneke, J.P.; Loftin, M.; Fukunaga, T. Prevalence of site-specific thigh sarcopenia in Japanese men and women. *Age (Dordrecht, Netherlands)* **2014**, *36*, 417-426, doi:10.1007/s11357-013-9539-6.
32. Abe, T.; Thiebaud, R.S.; Loenneke, J.P.; Ogawa, M.; Mitsukawa, N. Association between forearm muscle thickness and age-related loss of skeletal muscle mass, handgrip and knee extension strength and walking performance in old men and women: a pilot study. *Ultrasound in medicine & biology* **2014**, *40*, 2069-2075, doi:10.1016/j.ultrasmedbio.2014.05.003.
33. Maddocks, M.; Jones, M.; Snell, T.; Connolly, B.; de Wolf-Linder, S.; Moxham, J.; Rafferty, G.F. Ankle dorsiflexor muscle size, composition and force with ageing and chronic obstructive pulmonary disease. *Experimental physiology* **2014**, *99*, 1078-1088, doi:10.1113/expphysiol.2014.080093.
34. Souza, H.; Rocha, T.; Pessoa, M.; Rattes, C.; Brandão, D.; Fregonezi, G.; Campos, S.; Aliverti, A.; Dornelas, A. Effects of inspiratory muscle training in elderly women on respiratory muscle strength, diaphragm thickness and mobility. *The journals of gerontology. Series A, Biological sciences and medical sciences* **2014**, *69*, 1545-1553, doi:10.1093/gerona/glu182.
35. Takai, Y.; Ohta, M.; Akagi, R.; Kato, E.; Wakahara, T.; Kawakami, Y.; Fukunaga, T.; Kanehisa, H. Applicability of ultrasound muscle thickness measurements for predicting fat-free mass in elderly population. *The journal of nutrition, health & aging* **2014**, *18*, 579-585, doi:10.1007/s12603-013-0419-7.
36. Abe, T.; Counts, B.R.; Barnett, B.E.; Dankel, S.J.; Lee, K.; Loenneke, J.P. Associations between Handgrip Strength and Ultrasound-Measured Muscle Thickness of the Hand and Forearm in Young Men and Women. *Ultrasound in medicine & biology* **2015**, *41*, 2125-2130, doi:10.1016/j.ultrasmedbio.2015.04.004.
37. Abe, T.; Loenneke, J.P.; Young, K.C.; Thiebaud, R.S.; Nahar, V.K.; Hollaway, K.M.; Stover, C.D.; Ford, M.A.; Bass, M.A.; Loftin, M. Validity of ultrasound prediction equations for total and regional muscularity in middle-aged and older men and women. *Ultrasound in medicine & biology* **2015**, *41*, 557-564, doi:10.1016/j.ultrasmedbio.2014.09.007.
38. Berger, J.; Bunout, D.; Barrera, G.; de la Maza, M.P.; Henriquez, S.; Leiva, L.; Hirsch, S. Rectus femoris (RF) ultrasound for the assessment of muscle mass in older people. *Archives of Gerontology and Geriatrics* **2015**, *61*, 33-38, doi:https://doi.org/10.1016/j.archger.2015.03.006.
39. Greening, N.J.; Harvey-Dunstan, T.C.; Chaplin, E.J.; Vincent, E.E.; Morgan, M.D.; Singh, S.J.; Steiner, M.C. Bedside assessment of quadriceps muscle by ultrasound after admission for acute exacerbations of chronic respiratory disease. *American journal of respiratory and critical care medicine* **2015**, *192*, 810-816, doi:10.1164/rccm.201503-0535OC.
40. Ido, A.; Nakayama, Y.; Ishii, K.; Iemitsu, M.; Sato, K.; Fujimoto, M.; Kurihara, T.; Hamaoka, T.; Satoh-Asahara, N.; Sanada, K. Ultrasound-Derived Abdominal Muscle Thickness Better Detects Metabolic Syndrome Risk in Obese Patients than Skeletal Muscle Index Measured by Dual-Energy X-Ray Absorptiometry. *PloS one* **2015**, *10*, e0143858, doi:10.1371/journal.pone.0143858.
41. Ismail, C.; Zabal, J.; Hernandez, H.J.; Woletz, P.; Manning, H.; Teixeira, C.; DiPietro, L.; Blackman, M.R.; Harris-Love, M.O. Diagnostic ultrasound estimates of muscle mass and muscle quality discriminate between women with and without sarcopenia. *Frontiers in physiology* **2015**, *6*, 302, doi:10.3389/fphys.2015.00302.
42. Ando, R.; Nosaka, K.; Inami, T.; Tomita, A.; Watanabe, K.; Blazevich, A.J.; Akima, H. Difference in fascicle behaviors between superficial and deep quadriceps muscles during isometric contractions. *Muscle & nerve* **2016**, *53*, 797-802, doi:10.1002/mus.24905.
43. Carrillo-Esper, R.; Pérez-Calatayud Á, A.; Arch-Tirado, E.; Díaz-Carrillo, M.A.; Garrido-Aguirre, E.; Tapia-Velazco, R.; Peña-Pérez, C.A.; Espinoza-de Los Monteros, I.; Meza-Márquez, J.M.; Flores-Rivera, O.I., et al. Standardization of Sonographic Diaphragm Thickness Evaluations in Healthy Volunteers. *Respiratory care* **2016**, *61*, 920-924, doi:10.4187/respcare.03999.

44. Kuyumcu, M.E.; Halil, M.; Kara, Ö.; Çuni, B.; Çağlayan, G.; Güven, S.; Yeşil, Y.; Arık, G.; Yavuz, B.B.; Cankurtaran, M., et al. Ultrasonographic evaluation of the calf muscle mass and architecture in elderly patients with and without sarcopenia. *Archives of Gerontology and Geriatrics* **2016**, *65*, 218-224, doi:https://doi.org/10.1016/j.archger.2016.04.004.
45. Minetto, M.A.; Caresio, C.; Menapace, T.; Hajdarevic, A.; Marchini, A.; Molinari, F.; Maffiuletti, N.A. Ultrasound-Based Detection of Low Muscle Mass for Diagnosis of Sarcopenia in Older Adults. *PM&R* **2016**, *8*, 453-462, doi:https://doi.org/10.1016/j.pmrj.2015.09.014.
46. Mueller, N.; Murthy, S.; Tainter, C.R.; Lee, J.; Riddell, K.; Fintelmann, F.J.; Grabitz, S.D.; Timm, F.P.; Levi, B.; Kurth, T., et al. Can Sarcopenia Quantified by Ultrasound of the Rectus Femoris Muscle Predict Adverse Outcome of Surgical Intensive Care Unit Patients as well as Frailty? A Prospective, Observational Cohort Study. *Annals of surgery* **2016**, *264*, 1116-1124, doi:10.1097/sla.0000000000001546.
47. Tandon, P.; Low, G.; Mourtzakis, M.; Zenith, L.; Myers, R.P.; Abraldes, J.G.; Shaheen, A.A.; Qamar, H.; Mansoor, N.; Carbonneau, M., et al. A Model to Identify Sarcopenia in Patients With Cirrhosis. *Clinical gastroenterology and hepatology : the official clinical practice journal of the American Gastroenterological Association* **2016**, *14*, 1473-1480.e1473, doi:10.1016/j.cgh.2016.04.040.
48. Sipilä, S.; Suominen, H. Muscle ultrasonography and computed tomography in elderly trained and untrained women. *Muscle & nerve* **1993**, *16*, 294-300, doi:10.1002/mus.880160309.
49. Bemben, M.G. Use of diagnostic ultrasound for assessing muscle size. *Journal of strength and conditioning research* **2002**, *16*, 103-108.
50. Reeves, N.D.; Maganaris, C.N.; Narici, M.V. Ultrasonographic assessment of human skeletal muscle size. *European journal of applied physiology* **2004**, *91*, 116-118, doi:10.1007/s00421-003-0961-9.
51. MacGillivray, T.J.; Ross, E.; Simpson, H.A.; Greig, C.A. 3D freehand ultrasound for in vivo determination of human skeletal muscle volume. *Ultrasound in medicine & biology* **2009**, *35*, 928-935, doi:10.1016/j.ultrasmedbio.2008.11.013.
52. Stetts, D.M.; Freund, J.E.; Allison, S.C.; Carpenter, G. A rehabilitative ultrasound imaging investigation of lateral abdominal muscle thickness in healthy aging adults. *Journal of geriatric physical therapy (2001)* **2009**, *32*, 60-66, doi:10.1519/00139143-200932020-00004.
53. Staehli, S.; Glatthorn, J.F.; Casartelli, N.; Maffiuletti, N.A. Test-retest reliability of quadriceps muscle function outcomes in patients with knee osteoarthritis. *Journal of electromyography and kinesiology : official journal of the International Society of Electrophysiological Kinesiology* **2010**, *20*, 1058-1065, doi:10.1016/j.jelekin.2010.07.006.
54. English, C.K.; Thoirs, K.A.; Fisher, L.; McLennan, H.; Bernhardt, J. Ultrasound is a reliable measure of muscle thickness in acute stroke patients, for some, but not all anatomical sites: a study of the intra-rater reliability of muscle thickness measures in acute stroke patients. *Ultrasound in medicine & biology* **2012**, *38*, 368-376, doi:10.1016/j.ultrasmedbio.2011.12.012.
55. Raj, I.S.; Bird, S.R.; Shield, A.J. Reliability of ultrasonographic measurement of the architecture of the vastus lateralis and gastrocnemius medialis muscles in older adults. *Clinical physiology and functional imaging* **2012**, *32*, 65-70, doi:10.1111/j.1475-097X.2011.01056.x.
56. Thomaes, T.; Thomis, M.; Onkelinx, S.; Coudyzer, W.; Cornelissen, V.; Vanhees, L. Reliability and validity of the ultrasound technique to measure the rectus femoris muscle diameter in older CAD-patients. *BMC medical imaging* **2012**, *12*, 7, doi:10.1186/1471-2342-12-7.
57. Strasser, E.M.; Draskovits, T.; Praschak, M.; Quittan, M.; Graf, A. Association between ultrasound measurements of muscle thickness, pennation angle, echogenicity and skeletal muscle strength in the elderly. *Age (Dordrecht, Netherlands)* **2013**, *35*, 2377-2388, doi:10.1007/s11357-013-9517-z.
58. Takai, Y.; Ohta, M.; Akagi, R.; Kato, E.; Wakahara, T.; Kawakami, Y.; Fukunaga, T.; Kanehisa, H. Validity of ultrasound muscle thickness measurements for predicting leg skeletal muscle mass in healthy Japanese middle-aged and older individuals. *Journal of physiological anthropology* **2013**, *32*, 12, doi:10.1186/1880-6805-32-12.
59. Agyapong-Badu, S.; Warner, M.; Samuel, D.; Narici, M.; Cooper, C.; Stokes, M. Anterior thigh composition measured using ultrasound imaging to quantify relative thickness of muscle and non-contractile tissue: a potential biomarker for musculoskeletal health. *Physiological measurement* **2014**, *35*, 2165-2176, doi:10.1088/0967-3334/35/10/2165.
60. Cho, K.H.; Lee, H.J.; Lee, W.H. Reliability of rehabilitative ultrasound imaging for the medial gastrocnemius muscle in poststroke patients. *Clinical physiology and functional imaging* **2014**, *34*, 26-31, doi:10.1111/cpf.12060.
61. Hammond, K.; Mampilly, J.; Laghi, F.A.; Goyal, A.; Collins, E.G.; McBurney, C.; Jubran, A.; Tobin, M.J. Validity and reliability of rectus femoris ultrasound measurements: Comparison of curved-array and linear-array transducers. *Journal of rehabilitation research and development* **2014**, *51*, 1155-1164, doi:10.1682/jrrd.2013.08.0187.
62. Sions, J.M.; Velasco, T.O.; Teyhen, D.S.; Hicks, G.E. Ultrasound imaging: intraexaminer and interexaminer reliability for multifidus muscle thickness assessment in adults aged 60 to 85 years versus younger adults. *The Journal of orthopaedic and sports physical therapy* **2014**, *44*, 425-434, doi:10.2519/jospt.2014.4584.
63. Takai, Y.; Ohta, M.; Akagi, R.; Kato, E.; Wakahara, T.; Kawakami, Y.; Fukunaga, T.; Kanehisa, H. Applicability of ultrasound muscle thickness measurements for predicting fat-free mass in elderly population. *J Nutr Health Aging* **2014**, *18*, 579-585, doi:10.1007/s12603-013-0419-7.
64. Berger, J.; Bunout, D.; Barrera, G.; de la Maza, M.P.; Henriquez, S.; Leiva, L.; Hirsch, S. Rectus femoris (RF) ultrasound for the assessment of muscle mass in older people. *Arch Gerontol Geriatr* **2015**, *61*, 33-38, doi:10.1016/j.archger.2015.03.006.

65. Wang, C.-Z.; Li, T.-J.; Zheng, Y.-P. Shear modulus estimation on vastus intermedius of elderly and young females over the entire range of isometric contraction. *PloS one* **2014**, *9*, e101769-e101769, doi:10.1371/journal.pone.0101769.
66. Akagi, R.; Yamashita, Y.; Ueyasu, Y. Age-Related Differences in Muscle Shear Moduli in the Lower Extremity. *Ultrasound in medicine & biology* **2015**, *41*, 2906-2912, doi:10.1016/j.ultrasmedbio.2015.07.011.
67. Eby, S.F.; Cloud, B.A.; Brandenburg, J.E.; Giambini, H.; Song, P.; Chen, S.; LeBrasseur, N.K.; An, K.N. Shear wave elastography of passive skeletal muscle stiffness: influences of sex and age throughout adulthood. *Clinical biomechanics (Bristol, Avon)* **2015**, *30*, 22-27, doi:10.1016/j.clinbiomech.2014.11.011.
68. Heizelmann, A.; Tasdemir, S.; Schmidberger, J.; Gräter, T.; Kratzer, W.; Grüner, B. Measurements of the trapezius and erector spinae muscles using virtual touch imaging quantification ultrasound-Elastography: a cross section study. *BMC musculoskeletal disorders* **2017**, *18*, 370, doi:10.1186/s12891-017-1733-8.
69. Nakamura, M.; Ikezoe, T.; Nishishita, S.; Umehara, J.; Kimura, M.; Ichihashi, N. Acute effects of static stretching on the shear elastic moduli of the medial and lateral gastrocnemius muscles in young and elderly women. *Musculoskeletal science & practice* **2017**, *32*, 98-103, doi:10.1016/j.msksp.2017.09.006.
70. Wang, C.Z.; Guo, J.Y.; Li, T.J.; Zhou, Y.; Shi, W.; Zheng, Y.P. Age and Sex Effects on the Active Stiffness of Vastus Intermedius under Isometric Contraction. *BioMed research international* **2017**, *2017*, 9469548, doi:10.1155/2017/9469548.
71. Baumer, T.G.; Dischler, J.; Davis, L.; Labyed, Y.; Siegal, D.S.; van Holsbeeck, M.; Moutzouros, V.; Bey, M.J. Effects of age and pathology on shear wave speed of the human rotator cuff. *Journal of orthopaedic research : official publication of the Orthopaedic Research Society* **2018**, *36*, 282-288, doi:10.1002/jor.23641.
72. Alfuraih, A.M.; Tan, A.L.; O'Connor, P.; Emery, P.; Wakefield, R.J. The effect of ageing on shear wave elastography muscle stiffness in adults. *Aging clinical and experimental research* **2019**, *31*, 1755-1763, doi:10.1007/s40520-019-01139-0.
73. Saito, A.; Wakasa, M.; Kimoto, M.; Ishikawa, T.; Tsugaruya, M.; Kume, Y.; Okada, K. Age-related changes in muscle elasticity and thickness of the lower extremities are associated with physical functions among community-dwelling older women. *Geriatrics & gerontology international* **2019**, *19*, 61-65, doi:10.1111/ggi.13567.
74. Sun, Y.; Xiao, Y.; Li, F.; Wang, C.; Wu, T.; Zhou, M.; Cui, L. Diagnosing Muscle Atrophy by Use of a Comprehensive Method of Assessing the Elastic Properties of Muscle During Passive Stretching. *AJR. American journal of roentgenology* **2020**, *214*, 862-870, doi:10.2214/ajr.19.21174.
75. Vincent, M.A.; Clerk, L.H.; Lindner, J.R.; Price, W.J.; Jahn, L.A.; Leong-Poi, H.; Barrett, E.J. Mixed meal and light exercise each recruit muscle capillaries in healthy humans. *American journal of physiology. Endocrinology and metabolism* **2006**, *290*, E1191-1197, doi:10.1152/ajpendo.00497.2005.
76. Ikezoe, T.; Asakawa, Y.; Fukumoto, Y.; Tsukagoshi, R.; Ichihashi, N. Associations of muscle stiffness and thickness with muscle strength and muscle power in elderly women. *Geriatrics & gerontology international* **2012**, *12*, 86–92, doi:10.1111/j.1447-0594.2011.00735.x.
77. Abe, T.; Loenneke, J.P.; Thiebaud, R.S.; Fujita, E.; Akamine, T.; Loftin, M. Prediction and Validation of DXA-Derived Appendicular Fat-Free Adipose Tissue by a Single Ultrasound Image of the Forearm in Japanese Older Adults. *Journal of ultrasound in medicine : official journal of the American Institute of Ultrasound in Medicine* **2018**, *37*, 347–353, doi:10.1002/jum.14343.
78. Aboufazeli, M.; Afshar-Mohajer, N. Within-day and between-day reliability of thickness measurements of abdominal muscles using ultrasound during abdominal hollowing and bracing maneuvers. *Journal of bodywork and movement therapies* **2018**, *22*, 122–128, doi:10.1016/j.jbmt.2017.03.006.
79. Aboufazeli, M.; Akbari, M.; Jamshidi, A.A.; Jafarpisheh, M.S. Comparison of Selective Local and Global Muscle Thicknesses in Females with and without Chronic Low Back Pain. *Ortopedia, traumatologia, rehabilitacja* **2018**, *20*, 197–204, doi:10.5604/01.3001.0012.1473.
80. Akagi, R.; Suzuki, M.; Kawaguchi, E.; Miyamoto, N.; Yamada, Y.; Ema, R. Muscle size-strength relationship including ultrasonographic echo intensity and voluntary activation level of a muscle group. *Arch Gerontol Geriatr* **2018**, *75*, 185–190, doi:10.1016/j.archger.2017.12.012.
81. Akazawa, N.; Harada, K.; Okawa, N.; Tamura, K.; Hayase, A.; Moriyama, H. Relationships between muscle mass, intramuscular adipose and fibrous tissues of the quadriceps, and gait independence in chronic stroke survivors: a cross-sectional study. *Physiotherapy* **2018**, *104*, 438–445, doi:10.1016/j.physio.2017.08.009.
82. Allison, S.J.; Brooke-Wavell, K.; Folland, J. High and odd impact exercise training improved physical function and fall risk factors in community-dwelling older men. *Journal of musculoskeletal & neuronal interactions* **2018**, *18*, 100–107.
83. Alonso-Fernandez, D.; Gutierrez-Sanchez, Á.; Garcia-Remeseiro, T.; Garganta, R. Effects of the Nordic hamstring exercise on the architecture of the semitendinosus. **2018**.
84. Blue, M.N.M.; Smith-Ryan, A.E.; Trexler, E.T.; Hirsch, K.R. The effects of high intensity interval training on muscle size and quality in overweight and obese adults. *Journal of science and medicine in sport* **2018**, *21*, 207–212, doi:10.1016/j.jsams.2017.06.001.
85. Burton, A.M.; Stock, M.S. Consistency of novel ultrasound equations for estimating percent intramuscular fat. *Clinical physiology and functional imaging* **2018**, 10.1111/cpf.12532, doi:10.1111/cpf.12532.

86. Chang, K.V.; Wu, W.T.; Huang, K.C.; Jan, W.H.; Han, D.S. Limb muscle quality and quantity in elderly adults with dynapenia but not sarcopenia: An ultrasound imaging study. *Experimental gerontology* **2018**, *108*, 54–61, doi:10.1016/j.exger.2018.03.019.
87. Cheng, D.T.H.; Lee, K.Y.S.; Ahuja, A.T.; Tong, M.C.F. Sonographic assessment of swallowing in irradiated nasopharyngeal carcinoma patients. *The Laryngoscope* **2018**, *128*, 2552–2559, doi:10.1002/lary.27222.
88. de Lima, F.D.; Bottaro, M.; de Oliveira Valeriano, R.; Cruz, L.; Battaglini, C.L.; Vieira, C.A.; de Oliveira, R.J. Cancer-Related Fatigue and Muscle Quality in Hodgkin's Lymphoma Survivors. *Integrative cancer therapies* **2018**, *17*, 299–305, doi:10.1177/1534735417712009.
89. Dunford, E.C.; Au, J.S.; Devries, M.C.; Phillips, S.M.; MacDonald, M.J. Cardiovascular aging and the microcirculation of skeletal muscle: using contrast-enhanced ultrasound. *American journal of physiology. Heart and circulatory physiology* **2018**, *315*, H1194-h1199, doi:10.1152/ajpheart.00737.2017.
90. Emami, F.; Yoosefinejad, A.K.; Razeghi, M. Correlations between core muscle geometry, pain intensity, functional disability and postural balance in patients with nonspecific mechanical low back pain. *Medical engineering & physics* **2018**, *60*, 39–46, doi:10.1016/j.medengphy.2018.07.006.
91. Franchi, M.V.; Longo, S.; Mallinson, J.; Quinlan, J.I.; Taylor, T.; Greenhaff, P.L.; Narici, M.V. Muscle thickness correlates to muscle cross-sectional area in the assessment of strength training-induced hypertrophy. *Scandinavian journal of medicine & science in sports* **2018**, *28*, 846–853, doi:10.1111/sms.12961.
92. Fukumoto, Y.; Yamada, Y.; Ikezoe, T.; Watanabe, Y.; Taniguchi, M.; Sawano, S.; Minami, S.; Asai, T.; Kimura, M.; Ichihashi, N. Association of physical activity with age-related changes in muscle echo intensity in older adults: a 4-year longitudinal study. *Journal of applied physiology (Bethesda, Md. : 1985)* **2018**, *125*, 1468–1474, doi:10.1152/japplphysiol.00317.2018.
93. Gaffney, K.A.; Lucero, A.; Stoner, L.; Faulkner, J.; Whitfield, P.; Krebs, J.; Rowlands, D.S. Nil Whey Protein Effect on Glycemic Control after Intense Mixed-Mode Training in Type 2 Diabetes. *Medicine and science in sports and exercise* **2018**, *50*, 11–17, doi:10.1249/mss.0000000000001404.
94. Goodarzi, F.; Rahnama, L.; Karimi, N.; Baghi, R.; Jaberzadeh, S. The Effects of Forward Head Posture on Neck Extensor Muscle Thickness: An Ultrasonographic Study. *Journal of manipulative and physiological therapeutics* **2018**, *41*, 34–41, doi:10.1016/j.jmpt.2017.07.012.
95. Hadda, V.; Kumar, R.; Hussain, T.; Khan, M.A.; Madan, K.; Mohan, A.; Khilnani, G.C.; Guleria, R. Reliability of ultrasonographic arm muscle thickness measurement by various levels of health care providers in ICU. *Clinical nutrition ESPEN* **2018**, *24*, 78–81, doi:10.1016/j.clnesp.2018.01.009.
96. Harris-Love, M.O.; Avila, N.A.; Adams, B.; Zhou, J.; Seamon, B.; Ismail, C.; Zaidi, S.H.; Kassner, C.A.; Liu, F.; Blackman, M.R. The Comparative Associations of Ultrasound and Computed Tomography Estimates of Muscle Quality with Physical Performance and Metabolic Parameters in Older Men. *Journal of clinical medicine* **2018**, *7*, doi:10.3390/jcm7100340.
97. Hemelryck, W.; Calistri, J.; Papadopoulou, V.; Theunissen, S.; Dugardeyn, C.; Balestra, C. ULTRASONOGRAPHIC ASSESSMENT OF NECK MUSCULAR SIZE AND RANGE OF MOTION IN RUGBY PLAYERS. *International journal of sports physical therapy* **2018**, *13*, 28–38.
98. Hida, T.; Ando, K.; Kobayashi, K.; Ito, K.; Tsushima, M.; Kobayakawa, T.; Morozumi, M.; Tanaka, S.; Machino, M.; Ota, K., et al. ＜Editors' Choice＞ Ultrasound measurement of thigh muscle thickness for assessment of sarcopenia. *Nagoya journal of medical science* **2018**, *80*, 519–527, doi:10.18999/nagjms.80.4.519.
99. Holtzhausen, S.; Unger, M.; Lupton-Smith, A.; Hanekom, S. An investigation into the use of ultrasound as a surrogate measure of diaphragm function. *Heart & lung : the journal of critical care* **2018**, *47*, 418–424, doi:10.1016/j.hrtlng.2018.04.010.
100. Kajisa, E.; Tohara, H.; Nakane, A.; Wakasugi, Y.; Hara, K.; Yamaguchi, K.; Yoshimi, K.; Minakuchi, S. The relationship between jaw-opening force and the cross-sectional area of the suprahyoid muscles in healthy elderly. *Journal of oral rehabilitation* **2018**, *45*, 222–227, doi:10.1111/joor.12596.
101. Kato, E.; Nakamura, M.; Takahashi, H. Effect of Compression Garments on Controlled Force Output After Heel-Rise Exercise. *Journal of strength and conditioning research* **2018**, *32*, 1174–1179, doi:10.1519/jsc.0000000000001919.
102. Kawai, H.; Kera, T.; Hirayama, R.; Hirano, H.; Fujiwara, Y.; Ihara, K.; Kojima, M.; Obuchi, S. Morphological and qualitative characteristics of the quadriceps muscle of community-dwelling older adults based on ultrasound imaging: classification using latent class analysis. *Aging clinical and experimental research* **2018**, *30*, 283–291, doi:10.1007/s40520-017-0781-0.
103. Kim, D.K.; Kim, C.Y.; Lee, B.K.; Seo, D. A comparison of ultrasonography measurement on the abdominal muscle thickness between adolescent idiopathic scoliosis and healthy subjects. *Journal of back and musculoskeletal rehabilitation* **2018**, *31*, 65–74, doi:10.3233/bmr-169667.
104. Kruse, N.T.; Hughes, W.E.; Casey, D.P. Mechanistic insights into the modulatory role of the mechanoreflex on central hemodynamics using passive leg movement in humans. *Journal of applied physiology (Bethesda, Md. : 1985)* **2018**, *125*, 545–552, doi:10.1152/japplphysiol.01085.2017.
105. Kwan, B.C.H.; McBain, R.A.; Luu, B.L.; Butler, J.E.; Bilston, L.E.; Gandevia, S.C. Influence of respiratory mechanics and drive on genioglossus movement under ultrasound imaging. *PloS one* **2018**, *13*, e0195884, doi:10.1371/journal.pone.0195884.

106. Lee, H.J.; Ha, H.G.; Hahn, J.; Lim, S.; Lee, W.H. Intra- and inter-rater reliabilities for novel muscle thickness assessment during Co-contraction with dual-rehabilitative ultrasound imaging. *Physical therapy in sport : official journal of the Association of Chartered Physiotherapists in Sports Medicine* **2018**, *32*, 109–114, doi:10.1016/j.ptsp.2018.05.010.
107. Magrini, M.A.; Colquhoun, R.J.; Barrera-Curiel, A.; Thiele, R.M.; DeFreitas, J.M.; Smith, D.B.; Jenkins, N.D.M. Muscle size, strength, power, and echo intensity, but not specific tension, are affected by age in physically active adults. **2018**.
108. Mangum, L.C.; Henderson, K.; Murray, K.P.; Saliba, S.A. Ultrasound Assessment of the Transverse Abdominis During Functional Movement. *Journal of ultrasound in medicine : official journal of the American Institute of Ultrasound in Medicine* **2018**, *37*, 1225–1231, doi:10.1002/jum.14466.
109. McKenna, L.J.; de Ronde, M.; Le, M.; Burke, W.; Graves, A.; Williams, S.A. Measurement of muscle thickness of the serratus anterior and lower trapezius using ultrasound imaging in competitive recreational adult swimmers, with and without current shoulder pain. *Journal of science and medicine in sport* **2018**, *21*, 129–133, doi:10.1016/j.jsams.2017.06.022.
110. Merrigan, J.J.; White, J.B.; Hu, Y.E.; Stone, J.D.; Oliver, J.M.; Jones, M.T. Differences in elbow extensor muscle characteristics between resistance-trained men and women. *European journal of applied physiology* **2018**, *118*, 2359–2366, doi:10.1007/s00421-018-3962-4.
111. Michael, A.E.; Young, P. Is triceps hypertrophy associated with ulnar nerve luxation? *Muscle & nerve* **2018**, *58*, 523–527, doi:10.1002/mus.26183.
112. Mickle, K.J.; Nester, C.J. Morphology of the Toe Flexor Muscles in Older Adults With Toe Deformities. *Arthritis care & research* **2018**, *70*, 902–907, doi:10.1002/acr.23348.
113. Misirlioglu, T.O.; Ozyemisci Taskiran, O. Reliability of sonographic muscle thickness measurements of the thenar and hypothenar muscles. *Muscle & nerve* **2018**, *57*, E14-e17, doi:10.1002/mus.25735.
114. Murtagh, C.F.; Nulty, C.; Vanrenterghem, J.; O'Boyle, A.; Morgans, R.; Drust, B.; Erskine, R.M. The Neuromuscular Determinants of Unilateral Jump Performance in Soccer Players Are Direction-Specific. *International journal of sports physiology and performance* **2018**, *13*, 604–611, doi:10.1123/ijspp.2017-0589.
115. Nozoe, M.; Kanai, M.; Kubo, H.; Kobayashi, M.; Yamamoto, M.; Shimada, S.; Mase, K. Quadriceps muscle thickness changes in patients with aneurysmal subarachnoid hemorrhage during the acute phase. *Topics in stroke rehabilitation* **2018**, *25*, 209–213, doi:10.1080/10749357.2017.1413762.
116. Ogawa, N.; Mori, T.; Fujishima, I.; Wakabayashi, H.; Itoda, M.; Kunieda, K.; Shigematsu, T.; Nishioka, S.; Tohara, H.; Yamada, M., et al. Ultrasonography to Measure Swallowing Muscle Mass and Quality in Older Patients With Sarcopenic Dysphagia. *Journal of the American Medical Directors Association* **2018**, *19*, 516–522, doi:10.1016/j.jamda.2017.11.007.
117. Park, K.M.; Choi, E.; Kwak, E.J.; Kim, S.; Park, W.; Jeong, J.S.; Kim, K.D. The relationship between masseter muscle thickness measured by ultrasonography and facial profile in young Korean adults. *Imaging science in dentistry* **2018**, *48*, 213–221, doi:10.5624/isd.2018.48.3.213.
118. Poveda-Roda, R.; Moreno, P.; Bagán, J.; Margaix, M. Myofascial Pain: Ultrasound Width of the Masseter Muscle. *Journal of oral & facial pain and headache* **2018**, *32*, 298–303, doi:10.11607/ofph.1944.
119. Silva, C.R.S.; Costa, A.D.S.; Rocha, T.; de Lima, D.A.M.; do Nascimento, T.; de Moraes, S.R.A. Quadriceps muscle architecture ultrasonography of individuals with type 2 diabetes: Reliability and applicability. *PloS one* **2018**, *13*, e0205724, doi:10.1371/journal.pone.0205724.
120. Sterczala, A.J.; Herda, T.J.; Miller, J.D.; Ciccone, A.B.; Trevino, M.A. Age-related differences in the motor unit action potential size in relation to recruitment threshold. *Clinical physiology and functional imaging* **2018**, *38*, 610–616, doi:10.1111/cpf.12453.
121. Sutherlin, M.A.; Gage, M.; Mangum, L.C.; Hertel, J.; Russell, S.; Saliba, S.A.; Hart, J.M. Changes in Muscle Thickness Across Positions on Ultrasound Imaging in Participants With or Without a History of Low Back Pain. *Journal of athletic training* **2018**, *53*, 553–559, doi:10.4085/1062-6050-491-16.
122. Watanabe, Y.; Ikenaga, M.; Yoshimura, E.; Yamada, Y.; Kimura, M. Association between echo intensity and attenuation of skeletal muscle in young and older adults: a comparison between ultrasonography and computed tomography. *Clinical interventions in aging* **2018**, *13*, 1871–1878, doi:10.2147/cia.S173372.
123. Yoshiko, A.; Kaji, T.; Sugiyama, H.; Koike, T.; Oshida, Y.; Akima, H. Muscle quality characteristics of muscles in the thigh, upper arm and lower back in elderly men and women. *European journal of applied physiology* **2018**, *118*, 1385–1395, doi:10.1007/s00421-018-3870-7.
124. Akazawa, N.; Harada, K.; Okawa, N.; Tamura, K.; Moriyama, H. Low body mass index negatively affects muscle mass and intramuscular fat of chronic stroke survivors. *PloS one* **2019**, *14*, e0211145, doi:10.1371/journal.pone.0211145.
125. Alfuraih, A.M.; Tan, A.L.; O'Connor, P.; Emery, P.; Wakefield, R.J. The effect of ageing on shear wave elastography muscle stiffness in adults. *Aging clinical and experimental research* **2019**, *31*, 1755–1763, doi:10.1007/s40520-019-01139-0.
126. Aubertin-Leheudre, M.; Martel, D.; Narici, M.; Bonnefoy, M. The usefulness of muscle architecture assessed with ultrasound to identify hospitalized older adults with physical decline. *Experimental gerontology* **2019**, *125*, 110678, doi:10.1016/j.exger.2019.110678.
127. Bartolomei, S.; Rovai, C.; Lanzoni, I.M.; di Michele, R. Relationships Between Muscle Architecture, Deadlift Performance, and Maximal Isometric Force Produced at the Midthigh and Midshin Pull in Resistance-Trained Individuals. *Journal of strength and conditioning research* **2019**, 10.1519/jsc.0000000000003455, doi:10.1519/jsc.0000000000003455.

128. Bey, M.E.; Marzilger, R.; Hinkson, L.; Arampatzis, A.; Legerlotz, K. Vastus Lateralis Architecture Changes During Pregnancy - A Longitudinal Study. *Frontiers in physiology* **2019**, *10*, 1163, doi:10.3389/fphys.2019.01163.
129. Carletti, T.M.; Pinheiro, M.A.; Gonçalves, T.; Rodrigues Garcia, R.C.M. Influence of lower complete denture use on masseter muscles and masticatory function: A longitudinal study. *Journal of oral rehabilitation* **2019**, *46*, 127–133, doi:10.1111/joor.12729.
130. Chantaramanee, A.; Tohara, H.; Nakagawa, K.; Hara, K.; Nakane, A.; Yamaguchi, K.; Yoshimi, K.; Junichi, F.; Minakuchi, S. Association between echo intensity of the tongue and its thickness and function in elderly subjects. *Journal of oral rehabilitation* **2019**, *46*, 634–639, doi:10.1111/joor.12788.
131. Cuellar, W.A.; Blizzard, L.; Hides, J.A.; Callisaya, M.L.; Jones, G.; Cicuttini, F.; Wluka, A.E.; Ding, C.; Winzenberg, T.M. Vitamin D supplements for trunk muscle morphology in older adults: secondary analysis of a randomized controlled trial. *Journal of cachexia, sarcopenia and muscle* **2019**, *10*, 177–187, doi:10.1002/jcsm.12364.
132. Hasegawa, Y.; Yoshida, M.; Sato, A.; Fujimoto, Y.; Minematsu, T.; Sugama, J.; Sanada, H. Temporal muscle thickness as a new indicator of nutritional status in older individuals. *Geriatrics & gerontology international* **2019**, *19*, 135–140, doi:10.1111/ggi.13570.
133. Lopez, P.; Pinto, M.D.; Pinto, R.S. Does Rest Time before Ultrasonography Imaging Affect Quadriceps Femoris Muscle Thickness, Cross-Sectional Area and Echo Intensity Measurements? *Ultrasound in medicine & biology* **2019**, *45*, 612–616, doi:10.1016/j.ultrasmedbio.2018.10.010.
134. Saito, A.; Wakasa, M.; Kimoto, M.; Ishikawa, T.; Tsugaruya, M.; Kume, Y.; Okada, K. Age-related changes in muscle elasticity and thickness of the lower extremities are associated with physical functions among community-dwelling older women. *Geriatrics & gerontology international* **2019**, *19*, 61–65, doi:10.1111/ggi.13567.
135. Shah, S.P.; Penn, K.; Kaplan, S.J.; Vrablik, M.; Jablonowski, K.; Pham, T.N.; Reed, M.J. Comparison of bedside screening methods for frailty assessment in older adult trauma patients in the emergency department. *The American journal of emergency medicine* **2019**, *37*, 12–18, doi:10.1016/j.ajem.2018.04.028.
136. Šimunič, B.; Koren, K.; Rittweger, J.; Lazzer, S.; Reggiani, C.; Rejc, E.; Pišot, R.; Narici, M.; Degens, H. Tensiomyography detects early hallmarks of bed-rest-induced atrophy before changes in muscle architecture. *Journal of applied physiology (Bethesda, Md. : 1985)* **2019**, *126*, 815–822, doi:10.1152/japplphysiol.00880.2018.
